# Supplementary material for: A proximity-labeling-based approach to directly detect mRNA delivery to specific subcellular locations
Source: Mol Ther Nucleic Acids. 2025 Jun 24;36(3):102602. doi: 10.1016/j.omtn.2025.102602 (PMC12256308; doi:10.1016/j.omtn.2025.102602)
Supplement: Document S1. Figures S1–S5 and Tables S1 and S2 [file mmc1.pdf]

OMTN, Volume *36*

Supplemental information

A proximity-labeling-based approach to directly detect mRNA delivery to specific subcellular locations

Alfredo D. Smart, Merryn E. Hughes, Angela Downie Ruiz Velasco, Naoto Hori, Snow Stolnik, and Catherine L. Jopling

Supplemental Methods

APEX2-RT-qPCR without spike-in normalization
HEK293T APEX2-ERM or HEK293T APEX2-NES cells were transfected with Nluc or IL6-Nluc mRNA and APEX2 labelling carried out as described in the main methods. In early experiments before optimization of streptavidin pulldown or introduction of a biotinylated spike-in RNA, streptavidin pulldown was carried out as described in.[16] Nluc mRNA levels in pulldown RNA were measured by RT-qPCR relative to 2% input RNA, and are shown relative to a no-biotin control. As the Shapiro-Wilk test showed that data were not normally distributed, statistical comparison was carried out by Mann-Whitney test.

Table S1: Primer sequences. Primers used in PCR amplification for restriction cloning or Gibson assembly to generate plasmids, PCR amplification to generate *in vitro* transcription templates, and for RT-qPCR.

Primer name	Primer sequence 5'-3'	Purpose
HBB_Nluc_F	GATCGTCAAAGCTTACTTGGTC	Restriction cloning of Nluc and IL6-Nluc
HBB_Nluc_R	GATCTGACGAATTCTTGCAATG	
Nluc_alt_frag_F	CAGACACCATGGATCTGGTGCT	Gibson assembly of PMEL-Nluc
Nluc_alt_frag_R	TGTGAAGACTGTAGCCCCCACAGCCAG	
Amp_F	GCCATTGCTACAGGCATCGTGGTGTCACGC	
Nluc_alt_Vec_R	CAGATCCATGGTGTCTGTTTGAGGTTG	
Nluc_alt_Vec_F	GGGCTACAGTCTTCACACTCGAAGATTTCGT	
Amp_R	CTGTAGCAATGGCAACAACGTTGCGC	
PMEL_5'end_F	ACAGACACCATGGATCTGGTGCTAAAAAGATGCC	Gibson assembly of PMEL
PMEL_5'end_R	CACATAGCTCCGGGATCCCCGGC	
PMEL_mid_F	ATCCCGGAGCTATGTGCCTCTTGCTCATTCC	
PMEL_mid_R	CATACCTGTAGCCTCTGGAGTTGACATCTCTG	
PMEL_3'end_F	CCAGAGGCTACAGGTATGACACCTGCAGAGG	
PMEL_3'end_R	AAGCGAGCTCAGACCTGCTGCCCACTGA	
Amp_F	GCCATTGCTACAGGCATCGTGGTGTCACGC	
PMEL_Vec_R	CAGATCCATGGTGTCTGTTTGAGGT	
PMEL_Vec_F	GGTCTGAGCTCGCTTTCTTGC	
Amp_R	CTGTAGCAATGGCAACAACGTTGCGC	
OPMEL_IL6_Frag_F	CAGACACCATGGACTCCTTCTCC	Gibson assembly of IL6-PMEL
OPMEL_IL6_Frag_R	GGGCCTTTTGGGGCAGGGAAGGCA	
Amp_F	GCCATTGCTACAGGCATCGTGGTGTCACGC	
OPMEL_IL6_Vec_R	GGAGTCCATGGTGTCTGTTTGAGGTTGC	
OPMEL_IL6_Vec_F	TGCCCCAAAAGGCCCCAGAAACCAGG	
Amp_R	CTGTAGCAATGGCAACAACGTTGCGC	

HBB_IVT_F	GCCGCTAATACGACTCACTAT	IVT template
HBB_IVT_R	TTGCAATGAAAATAAATGTTTTTTATTAGGCAGAATCCAGA	
APEX_F	TTCGGAACCATCAAGCACCC	RT-qPCR of APEX2 mRNA
APEX_R	CCAGGGTGGAATGGAACCTTAG	
18S_F	CAGCCACCCGAGATTGAGCA	RT-qPCR of 18S rRNA
18S_R	TAGTAGCGACGGGCGGTGTG	
Nluc_F	CCGTATGAAGGTCTGAGCGG	RT-qPCR of Nluc mRNA
Nluc_R	TCTTTTTGCCGTCGAACACG	
Fluc_F	CTAAGGAAGTCGGGGAAGCG	RT-qPCR of Fluc mRNA
Fluc_R	ATCCCCCTCGGGTGTAATCA	
PMEL_F	TTAAGGCTGGTGAAGAGACAAG	RT-qPCR of PMEL mRNA
PMEL_R	AGGATCTCGGCACTTTCAATAC	

Table S2: mRNA sequences. Sequences of Nluc and PMEL mRNAs are shown in 5'-3' direction, with HBB 5' and 3'UTRs in lower case, coding sequence in upper case, and signal peptide coding sequence in bold. All mRNAs also included a 5' cap and a 3' enzymatically added pA tail.

**Nluc mRNA**
gggacatttgcttctgacacaactgtgttcactagcaacctcaaacagacaccATGGTCTTCACACTCGAAGATTTCGTTGGGGACTGGCGACAGACAGCCGGCTACAACCTGGACCAAGTCCTTGAACAGGGAGGTGTGTCCAGTTTGTTTCAGAATCTCGGGGTGTCCGTAACTCCGATCCAAAGGATTGTCCTGAGCGGTGAAAATGGGCTGAAGATCGACATCCATGTCATCATCCCGTATGAAGGTCTGAGCGGCGACCAAATGGGCCAGATCGAAAAAATTTTTAAGGTGGTGTACCCTGTGGATGATCATCACTTTAAGGTGATCCTGCACTATGGCACACTGGTAATCGACGGGGTTACGCCGAACATGATCGACTATTTCGGACGGCCGTATGAAGGCATCGCCGTGTTCGACGGCAAAAAGATCACTGTAACAGGGACCCTGTGGAACGGCAACAAAATTATCGACGAGCGCCTGATCAACCCCGACGGCTCCCTGCTGTTCCGAGTAACCATCAACGGAGTGACCGGCTGGCGGCTGTGCGAACGCATTCTGGCGTAAgctcgctttcttgctgtccaatttctattaaaggttcctttgttccctaagtccaactactaaactgggggatattatgaagggccttgagcatctggattctgcctaataaaaaacatttattttcattgcaa
**IL6-Nluc mRNA**
gggacatttgcttctgacacaactgtgttcactagcaacctcaaacagacacc**ATGGACTCCTTCTCCACAAGCGCCTTCGGTCCAGTTGCCTTCTCCCTGGGCCTGCTCCTGGTGTTGCCTGCTGCCTTCCCTGCCCCA**GTCTTCACACTCGAAGATTTCGTTGGGGACTGGCGACAGACAGCCGGCTACAACCTGGACCAAGTCCTTGAACAGGGAGGTGTGTCCAGTTTGTTTCAGAATCTCGGGGTGTCCGTAACTCCGATCCAAAGGATTGTCCTGAGCGGTGAAAATGGGCTGAAGATCGACATCCATGTCATCATCCCGTATGAAGGTCTGAGCGGCGACCAAATGGGCCAGATCGAAAAAATTTTTAAGGTGGTGTACCCTGTGGATGATCATCACTTTAAGGTGATCCTGCACTATGGCACACTGGTAATCGACGGGGTTACGCCGAACATGATCGACTATTTCGGACGGCCGTATGAAGGCATCGCCGTGTTCGACGGCAAAAAGATCACTGTAACAGGGACCCTGTGGAACGGCAACAAAATTATCGACGAGCGCCTGATCAACCCCGACGGCTCCCTGCTGTTCCGAGTAACCATCAACGGAGTGACCGGCTGGCGGCTGTGCGAACGCATTCTGGCGTAAgctcgctttcttgctgtccaatttctattaaaggttc

ctttgttccctaagtccaactactaaactgggggatattatgaagggccttgagcatctggattctgcc<br>taataaaaaacatttattttcattgcaa
**PMEL-Nluc mRNA**
gggacatttgcttctgacacaactgtgttcactagcaacctcaaacagacacc**ATGGATCTGGTGCTAA**<br>**AAAGATGCCTTCTTCATTTGGCTGTGATAGGTGCTTTGCTGGCTGTGGGGGCTACA**GTCTTCACACTCG<br>AAGATTTCGTTGGGGACTGGCGACAGACAGCCGGCTACAACCTGGACCAAGTCCTTGAACAGGGAGGTG<br>TGTCCAGTTTGTTTCAGAATCTCGGGGTGTCCGTAACTCCGATCCAAAGGATTGTCCTGAGCGGTGAAA<br>ATGGGCTGAAGATCGACATCCATGTCATCATCCCGTATGAAGGTCTGAGCGGCGACCAAATGGGCCAGA<br>TCGAAAAAATTTTTAAGGTGGTGTACCCTGTGGATGATCATCACTTTAAGGTGATCCTGCACTATGGCA<br>CACTGGTAATCGACGGGGTTACGCCGAACATGATCGACTATTTCGGACGGCCGTATGAAGGCATCGCCG<br>TGTTCGACGGCAAAAAGATCACTGTAACAGGGACCCTGTGGAACGGCAACAAAATTATCGACGAGCGCC<br>TGATCAACCCCGACGGCTCCCTGCTGTTCCGAGTAACCATCAACGGAGTGACCGGCTGGCGGCTGTGCG<br>AACGCATTCTGGCGTAAgctcgctttcttgctgtccaatttctattaaaggttcctttgttccctaagt<br>ccaactactaaactgggggatattatgaagggccttgagcatctggattctgcctaataaaaaacattt<br>attttcattgcaa
**PMEL mRNA**
gggacatttgcttctgacacaactgtgttcactagcaacctcaaacagacacc**ATGGATCTGGTGCTAA**<br>**AAAGATGCCTTCTTCATTTGGCTGTGATAGGTGCTTTGCTGGCTGTGGGGGCTACA**AAAGGCCCCAGAA<br>ACCAGGACTGGCTTGGTGTCTCAAGGCAACTCAGAACCAAAGCCTGGAACAGGCAGCTGTATCCAGAGT<br>GGACAGAAGCCCAGAGACTTGACTGCTGGAGAGGTGGTCAAGTGTCCCTCAAGGTCAGTAATGATGGGC<br>CTACACTGATTGGTGCAAATGCCTCCTTCTCTATTGCCTTGAACTTCCCTGGAAGCCAAAAGGTATTGC<br>CAGATGGGCAGGTTATCTGGGTCAACAATACCATCATCAATGGGAGCCAGGTGTGGGGAGGACAGCCAG<br>TGTATCCCCAGGAAACTGACGATGCCTGCATCTTCCCTGATGGTGGACCTTGCCCATCTGGCTCTTGGT<br>CTCAGAAGAGAAGCTTTGTTTATGTCTGGAAGACCTGGGGCCAATACTGGCAAGTTCTAGGGGGCCCAG<br>TGTCTGGGCTGAGCATTGGGACAGGCAGGGCAATGCTGGGCACACACACCATGGAAGTGACTGTCTACC<br>ATCGCCGGGGATCCCGGAGCTATGTGCCTCTTGCTCATTCCAGCTCAGCCTTCACCATTACTGACCAGG<br>TGCCTTTCTCCGTGAGCGTGTCCCAGTTGCGGGCCTTGGATGGAGGGAACAAGCACTTCCTGAGAAATC<br>AGCCTCTGACCTTTGCCCTCCAGCTCCATGACCCCAGTGGCTATCTGGCTGAAGCTGACCTCTCCTACA<br>CCTGGGACTTTGGAGACAGTAGTGGAACCCTGATCTCTCGGGCACTTGTGGTCACTCATACTTACCTGG<br>AGCCTGGCCCAGTCACTGCCCAGGTGGTCCTGCAGGCTGCCATTCCTCTCACCTCCTGTGGCTCCTCCC<br>CAGTTCCAGGCACCACAGATGGGCACAGGCCAACTGCAGAGGCCCCTAACACCACAGCTGGCCAAGTGC<br>CTACTACAGAAGTTGTGGGTACTACACCTGGTCAGGCGCCAACTGCAGAGCCCTCTGGAACCACATCTG<br>TGCAGGTGCCAACCACTGAAGTCATAAGCACTGCACCTGTGCAGATGCCAACTGCAGAGAGCACAGGTA<br>TGACACCTGAGAAGGTGCCAGTTTCAGAGGTCATGGGTACCACACTGGCAGAGATGTCAACTCCAGAGG<br>CTACAGGTATGACACCTGCAGAGGTATCAATTGTGGTGCTTTCTGGAACCACAGCTGCACAGGTAACAA<br>CTACAGAGTGGGTGGAGACCACAGCTAGAGAGCTACCTATCCCTGAGCCTGAAGGTCCAGATGCCAGCT<br>CAATCATGTCTACGGAAAGTATTACAGGTTCCCTGGGCCCCCTGCTGGATGGTACAGCCACCTTAAGGC<br>TGGTGAAGAGACAAGTCCCCCTGGATTGTGTTCTGTATCGATATGGTTCCTTTTCCGTCACCCTGGACA<br>TTGTCCAGGGTATTGAAAGTGCCGAGATCCTGCAGGCTGTGCCGTCCGGTGAGGGGGATGCATTTGAGC<br>TGACTGTGTCCTGCCAAGGCGGGCTGCCCAAGGAAGCCTGCATGGAGATCTCATCGCCAGGGTGCCAGC<br>CCCCTGCCCAGCGGCTGTGCCAGCCTGTGCTACCCAGCCCAGCCTGCCAGCTGGTTCTGCACCAGATAC<br>TGAAGGGTGGCTCGGGGACATACTGCCTCAATGTGTCTCTGGCTGATACCAACAGCCTGGCAGTGGTCA<br>GCACCCAGCTTATCATGCCTGGTCAAGAAGCAGGCCTTGGGCAGGTTCCGCTGATCGTGGGCATCTTGC<br>TGGTGTTGATGGCTGTGGTCCTTGCATCTCTGATATATAGGCGCAGACTTATGAAGCAAGACTTCTCCG<br>TACCCCAGTTGCCACATAGCAGCAGTCACTGGCTGCGTCTACCCCGCATCTTCTGCTCTTGTCCCATTG<br>GTGAGAACAGCCCCCTCCTCAGTGGGCAGCAGGTCTGAgctcgctttcttgctgtccaatttctattaa

aggttcctttgttccctaagtccaactactaaactgggggatattatgaagggccttgagcatctggat<br>tctgcctaataaaaaacatttattttcattgcaa
**IL6-PMEL mRNA**
gggacatttgcttctgacacaactgtgttcactagcaacctcaaacagacacc**ATGGACTCCTTCTCCA**<br>**CAAGCGCCTTCGGTCCAGTTGCCTTCTCCCTGGGCCTGCTCCTGGTGTTGCCTGCTGCCTTCCCTGCCC**<br>**CA**AAAGGCCCCAGAAACCAGGACTGGCTTGGTGTCTCAAGGCAACTCAGAACCAAAGCCTGGAACAGGC<br>AGCTGTATCCAGAGTGGACAGAAGCCCAGAGACTTGACTGCTGGAGAGGTGGTCAAGTGTCCCTCAAGG<br>TCAGTAATGATGGGCCTACACTGATTGGTGCAAATGCCTCCTTCTCTATTGCCTTGAACTTCCCTGGAA<br>GCCAAAAGGTATTGCCAGATGGGCAGGTTATCTGGGTCAACAATACCATCATCAATGGGAGCCAGGTGT<br>GGGGAGGACAGCCAGTGTATCCCCAGGAAACTGACGATGCCTGCATCTTCCCTGATGGTGGACCTTGCC<br>CATCTGGCTCTTGGTCTCAGAAGAGAAGCTTTGTTTATGTCTGGAAGACCTGGGGCCAATACTGGCAAG<br>TTCTAGGGGGCCCAGTGTCTGGGCTGAGCATTGGGACAGGCAGGGCAATGCTGGGCACACACACCATGG<br>AAGTGACTGTCTACCATCGCCGGGGATCCCGGAGCTATGTGCCTCTTGCTCATTCCAGCTCAGCCTTCA<br>CCATTACTGACCAGGTGCCTTTCTCCGTGAGCGTGTCCCAGTTGCGGGCCTTGGATGGAGGGAACAAGC<br>ACTTCCTGAGAAATCAGCCTCTGACCTTTGCCCTCCAGCTCCATGACCCCAGTGGCTATCTGGCTGAAG<br>CTGACCTCTCCTACACCTGGGACTTTGGAGACAGTAGTGGAACCCTGATCTCTCGGGCACTTGTGGTCA<br>CTCATACTTACCTGGAGCCTGGCCCAGTCACTGCCCAGGTGGTCCTGCAGGCTGCCATTCCTCTCACCT<br>CCTGTGGCTCCTCCCCAGTTCCAGGCACCACAGATGGGCACAGGCCAACTGCAGAGGCCCCTAACACCA<br>CAGCTGGCCAAGTGCCTACTACAGAAGTTGTGGGTACTACACCTGGTCAGGCGCCAACTGCAGAGCCCT<br>CTGGAACCACATCTGTGCAGGTGCCAACCACTGAAGTCATAAGCACTGCACCTGTGCAGATGCCAACTG<br>CAGAGAGCACAGGTATGACACCTGAGAAGGTGCCAGTTTCAGAGGTCATGGGTACCACACTGGCAGAGA<br>TGTCAACTCCAGAGGCTACAGGTATGACACCTGCAGAGGTATCAATTGTGGTGCTTTCTGGAACCACAG<br>CTGCACAGGTAACAACTACAGAGTGGGTGGAGACCACAGCTAGAGAGCTACCTATCCCTGAGCCTGAAG<br>GTCCAGATGCCAGCTCAATCATGTCTACGGAAAGTATTACAGGTTCCCTGGGCCCCCTGCTGGATGGTA<br>CAGCCACCTTAAGGCTGGTGAAGAGACAAGTCCCCCTGGATTGTGTTCTGTATCGATATGGTTCCTTTT<br>CCGTCACCCTGGACATTGTCCAGGGTATTGAAAGTGCCGAGATCCTGCAGGCTGTGCCGTCCGGTGAGG<br>GGGATGCATTTGAGCTGACTGTGTCCTGCCAAGGCGGGCTGCCCAAGGAAGCCTGCATGGAGATCTCAT<br>CGCCAGGGTGCCAGCCCCCTGCCCAGCGGCTGTGCCAGCCTGTGCTACCCAGCCCAGCCTGCCAGCTGG<br>TTCTGCACCAGATACTGAAGGGTGGCTCGGGGACATACTGCCTCAATGTGTCTCTGGCTGATACCAACA<br>GCCTGGCAGTGGTCAGCACCCAGCTTATCATGCCTGGTCAAGAAGCAGGCCTTGGGCAGGTTCCGCTGA<br>TCGTGGGCATCTTGCTGGTGTTGATGGCTGTGGTCCTTGCATCTCTGATATATAGGCGCAGACTTATGA<br>AGCAAGACTTCTCCGTACCCCAGTTGCCACATAGCAGCAGTCACTGGCTGCGTCTACCCCGCATCTTCT<br>GCTCTTGTCCCATTGGTGAGAACAGCCCCCTCCTCAGTGGGCAGCAGGTCTGAgctcgctttcttgctg<br>tccaatttctattaaaggttcctttgttccctaagtccaactactaaactgggggatattatgaagggc<br>cttgagcatctggattctgcctaataaaaaacatttattttcattgcaa

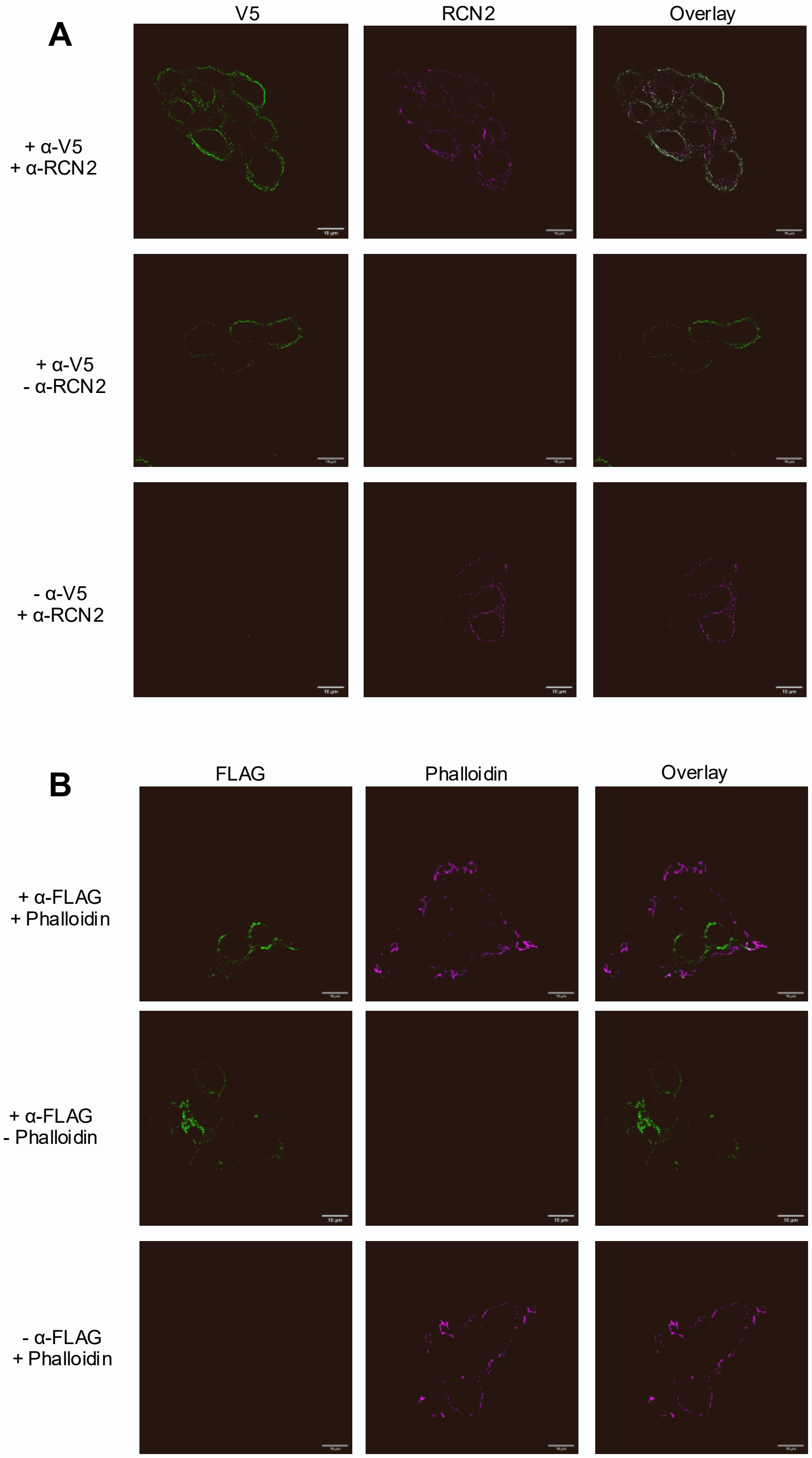

Figure S1. APEX2 localization in HEK293T APEX2-ERM and APEX2-NES cells. (A) SIM of HEK293T APEX2-ERM cells shows overlap between APEX2 and the ER, detected with antibodies to the V5 epitope and RCN2 respectively. Manders' Colocalization Coefficients were $M_1 = 0.6233 \pm 0.04986$ (V5 overlap with RCN2), $M_2 = 0.6701 \pm 0.08678$ (RCN2 overlap with V5), calculated from 17 cells. (B) SIM of HEK293T APEX2-NES cells shows some overlap between APEX2 and filamentous actin as a cytoplasmic marker, detected by an antibody to the FLAG epitope and phalloidin, respectively. Manders' Colocalization Coefficients were $M_1 = 0.6989 \pm 0.1855$ (FLAG overlap with phalloidin), $M_2 = 0.1120 \pm 0.1141$ (phalloidin overlap with FLAG), calculated from 15 cells. Scale bars represent 10 μm.

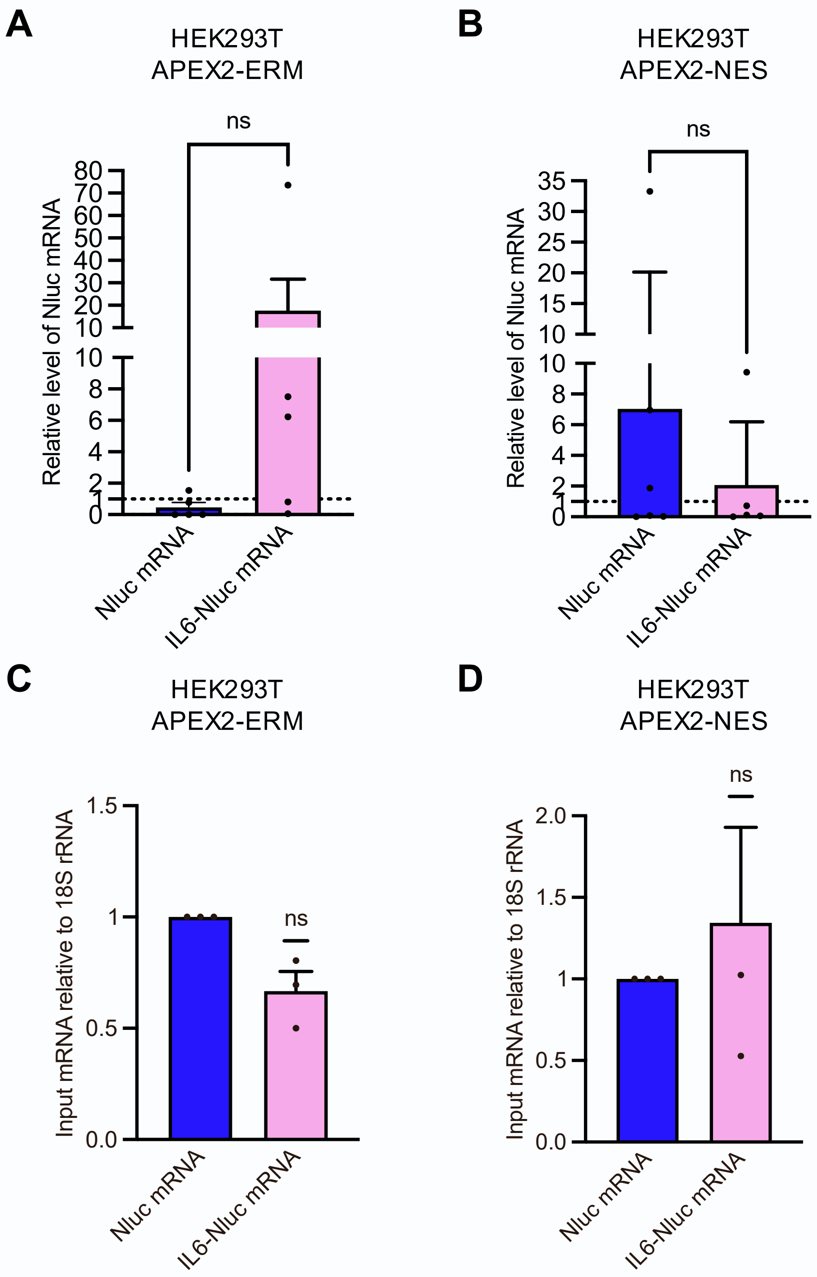

Figure S2. APEX2-RT-qPCR comparison of Nluc mRNA delivery to ER and cytoplasm. (A) HEK293T APEX2-ERM cells were transfected with Nluc or IL6-Nluc mRNA and APEX2 biotinylation and streptavidin pulldown carried out without inclusion of a spike-in RNA. Nluc mRNA in the pulldown measured by RT-qPCR was normalized to 2% input RNA and shown relative to a no-biotin control. Data represent mean of 5 independent experiments, +SEM. ns=not significant (Mann-Whitney test comparison of Nluc and IL6-Nluc). (B) As (A), except that HEK293T APEX2-NES cells were used. Data represent mean of 6 (Nluc) and 5 (IL6-Nluc) independent experiments, +SEM. ns=not significant (Mann-Whitney test comparison of Nluc and IL6-Nluc). (C) HEK293T APEX2-ERM cells were transfected with Nluc or IL6-Nluc mRNA for experiments shown in Figure 2G. Nluc mRNA was measured by RT-qPCR in input mRNA before streptavidin pulldown and show relative to 18S rRNA. Data represent mean of 3 independent experiments, +SEM. ns=not significant (Student's t test comparison of Nluc and IL6-Nluc). (D) As (C), except that HEK293T APEX2-NES cells were transfected for experiment shown in Figure 2H. Data represent mean of 3 independent experiments, +SEM. ns=not significant (Student's t test comparison of Nluc and IL6-Nluc).

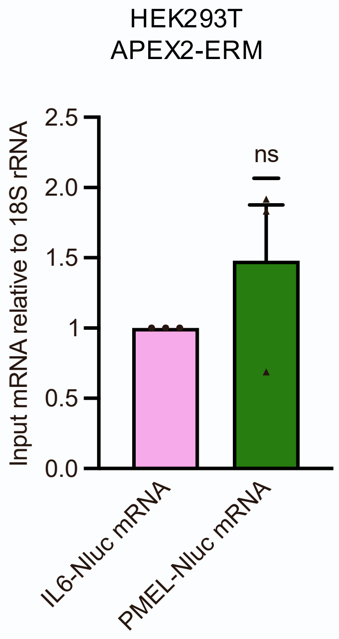

Figure S3. Input mRNA levels for HEK293T APEX2-ERM pulldown experiment. (A) HEK293T APEX2-ERM cells were transfected with IL6-Nluc or PMEL-Nluc mRNA for experiments shown in Figure 3B. Nluc mRNA was measured by RT-qPCR in input mRNA before streptavidin pulldown and show relative to 18S rRNA. Data represent mean of 3 independent experiments, +SEM. ns=not significant (Student's t test comparison of IL6-Nluc and PMEL-Nluc).

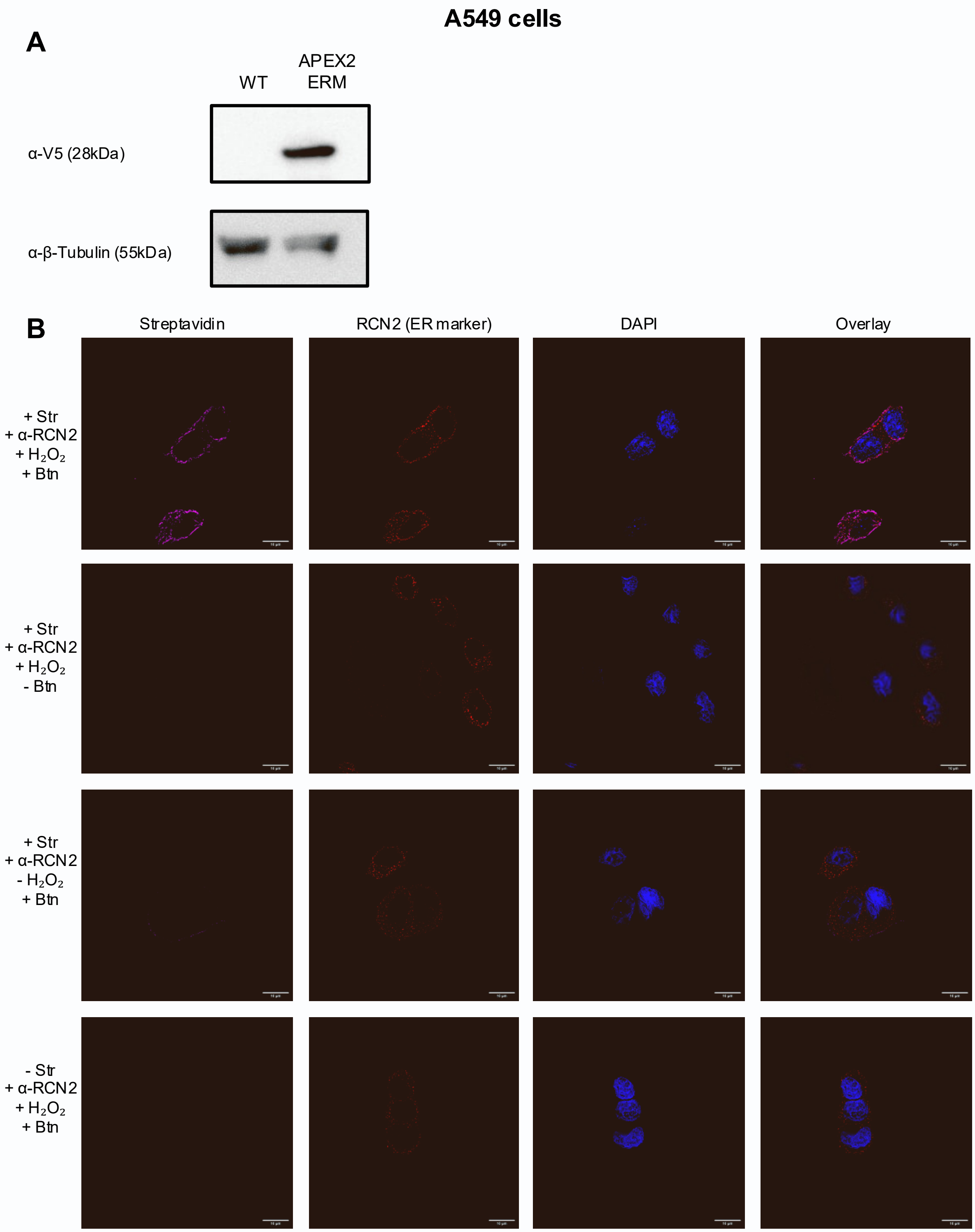

Figure S4. Characterization of A549 APEX2-ERM cells. (A) Western blot showing expression of APEX2-ERM (detected by antibody to V5 epitope) in an A549 cell line generated by lentiviral transduction. The membrane was stripped and re-probed for β-tubulin as a loading control. Image is representative of n=3 independent experiments. (B) SIM of HEK293T APEX2-ERM cells following an APEX2-mediated biotinylation reaction shows overlap between biotin and the ER, detected by streptavidin-AlexaFluor and anti-RCN2 respectively. DAPI is included as a nuclear stain. Manders' Colocalization Coefficients were $M_1 = 0.5584 \pm 0.05264$ (streptavidin overlap with RCN2), $M_2 = 0.8908 \pm 0.08908$ (RCN2 overlap with streptavidin), calculated from 25 cells. Scale bars represent 10 μm.

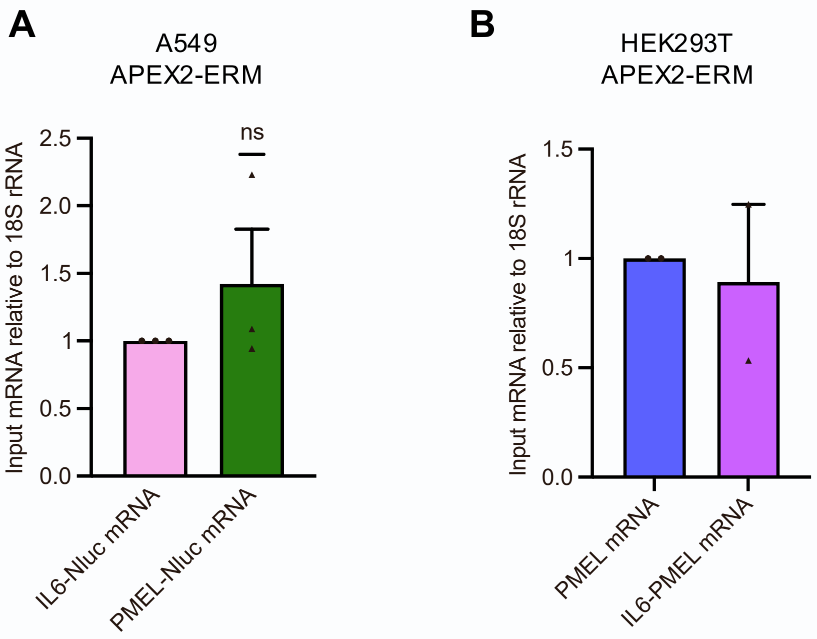

Figure S5. Input mRNA levels for APEX2 pulldown experiments. (A) A549 APEX2-ERM cells were transfected with IL6-Nluc or PMEL-Nluc mRNA for the experiments shown in Figure 3D. Nluc mRNA was measured by RT-qPCR in input mRNA before streptavidin pulldown and show relative to 18S rRNA. Data represent mean of 3 independent experiments, +SEM. ns=not significant (Student's t test comparison of IL6-Nluc and PMEL-Nluc). (B) As (A), except that HEK293T APEX2-ERM cells were transfected with PMEL or IL6-PMEL mRNA for the experiments shown in Figure 3G. Data represent mean of 2 independent experiments +SEM.
