# Supplementary material for: A proximity-labeling-based approach to directly detect mRNA delivery to specific subcellular locations
Source: Mol Ther Nucleic Acids. 2025 Jun 24;36(3):102602. doi: 10.1016/j.omtn.2025.102602 (PMC12256308; doi:10.1016/j.omtn.2025.102602)
Supplement: Document S2. Article plus supplemental information [file mmc2.pdf]

Molecular Therapy
Nucleic Acids
Brief report

A proximity-labeling-based approach to directly detect mRNA delivery to specific subcellular locations

Alfredo D. Smart,[1,2] Merryn E. Hughes,[1] Angela Downie Ruiz Velasco,[1] Naoto Hori,[1] Snow Stolnik,[1] and Catherine L. Jopling[1]

[1]School of Pharmacy, University of Nottingham, NG7 2RD Nottingham, UK

Messenger RNA (mRNA) therapeutics show considerable promise but face delivery challenges, as effective cytosolic entry and subsequent translation are normally limited by endosomal entrapment. While various approaches have been used to investigate determinants of effective RNA delivery, these methods tend to be indirect, qualitative, or rely on labeled RNA. There is a need for quantitative approaches that can directly measure mRNA delivery to its functional sites within the cell. Here, we adapted the APEXseq approach for proximity biotinylation and isolation of mRNA at specific subcellular locations. We combined APEX2 labeling with reverse-transcription quantitative PCR to investigate mRNA delivery to the cytoplasm and endoplasmic reticulum, the two major sites of translation, and found it was most effective in the endoplasmic reticulum. We incorporated a biotinylated spike-in RNA to improve existing methodology by allowing normalization of data and optimization of mRNA pull-down conditions. Finally, we combined this method with protein assays to investigate the role of different signal peptides in mRNA delivery to, and translation at, the endoplasmic reticulum. This new approach shows promise as a tool for future investigation of productive delivery of therapeutic mRNA.

INTRODUCTION

To maximize the potential of mRNA therapeutics, optimization of delivery platforms and mRNA design are necessary.[1,2] Current methods to investigate RNA intracellular delivery are generally based on imaging of fluorescently labeled lipid nanoparticles (LNPs) or RNA, which can be difficult to quantify, or on detection of encoded protein product, which is indirect.[3] Moreover, efficiency of cellular uptake does not always correlate with efficiency of encoded protein production,[4] and there has been little investigation and quantification of how delivered mRNA traffics within the cell and engages with the translation machinery.[2] mRNA subcellular localization can be important for correct protein production.[5] mRNA localization to the endoplasmic reticulum (ER) is required for production of secreted and membrane proteins and is therefore particularly relevant to mRNA therapeutics and vaccines.[6] ER targeting of mRNAs is usually mediated by cotranslational association between the signal recognition particle (SRP) and a signal peptide in the nascent chain of the protein emerging from the translating ribosome.[7] Signal peptides are diverse in sequence, although they have common structural features.[8]

Proximity biotinylation methods target enzymes that biotinylate neighboring proteins to locations within a cell, followed by streptavidin isolation of biotinylated proteins.[9,10] In contrast to commonly used methods such as BioID and TurboID,[11,12] the engineered peroxidase APEX2[13] also directly biotinylates RNA molecules, which can then be analyzed by RNA sequencing (RNA-seq) in the APEXseq method.[14] APEX2 labeling involves a short-lived (<1 ms) radical, allowing rapid biotinylation with a tight spatial radius.[13]

Here, we developed an improved protocol combining APEX2 labeling with reverse-transcription quantitative PCR (RT-qPCR), using a biotinylated spike-in RNA to optimize conditions and allow data normalization. We showed that this protocol can be used to investigate, and in principle provide quantification of, subcellular localization of *in vitro* transcribed mRNA (IVT mRNA) following its delivery to cells. We applied this method to investigate the effect of the signal peptide on mRNA delivery to the ER,[7] demonstrating its potential to provide new insight into therapeutic mRNA delivery.

RESULTS

We used lentiviral transduction to generate two HEK293T APEX2 cell lines. The first expresses APEX2 fused to an N-terminal signal peptide that mediates ER-targeting and to a C-terminal V5 epitope tag (APEX2-ERM).[14] The second expresses APEX2 fused to a C-terminal nuclear export signal to mediate cytoplasmic localization and to an N-terminal FLAG epitope tag (APEX2-NES).[14] We confirmed that APEX2 protein was expressed in both HEK293T APEX2-ERM and HEK293T APEX2-NES cell lines (Figure 1A), and *APEX2*

Received 18 March 2025; accepted 7 June 2025;
https://doi.org/10.1016/j.omtn.2025.102602.

[2]Present address: Department of Chemical Engineering and Biotechnology, University of Cambridge, Cambridge, UK

Correspondence: Catherine L. Jopling, School of Pharmacy, University of Nottingham, NG7 2RD Nottingham, UK.
E-mail: catherine.jopling@nottingham.ac.uk

© 2025 The Author(s).
Published by Elsevier Inc. on behalf of The American Society of Gene and Cell Therapy.
This is an open access article under the CC BY license (http://creativecommons.org/licenses/by/4.0/).

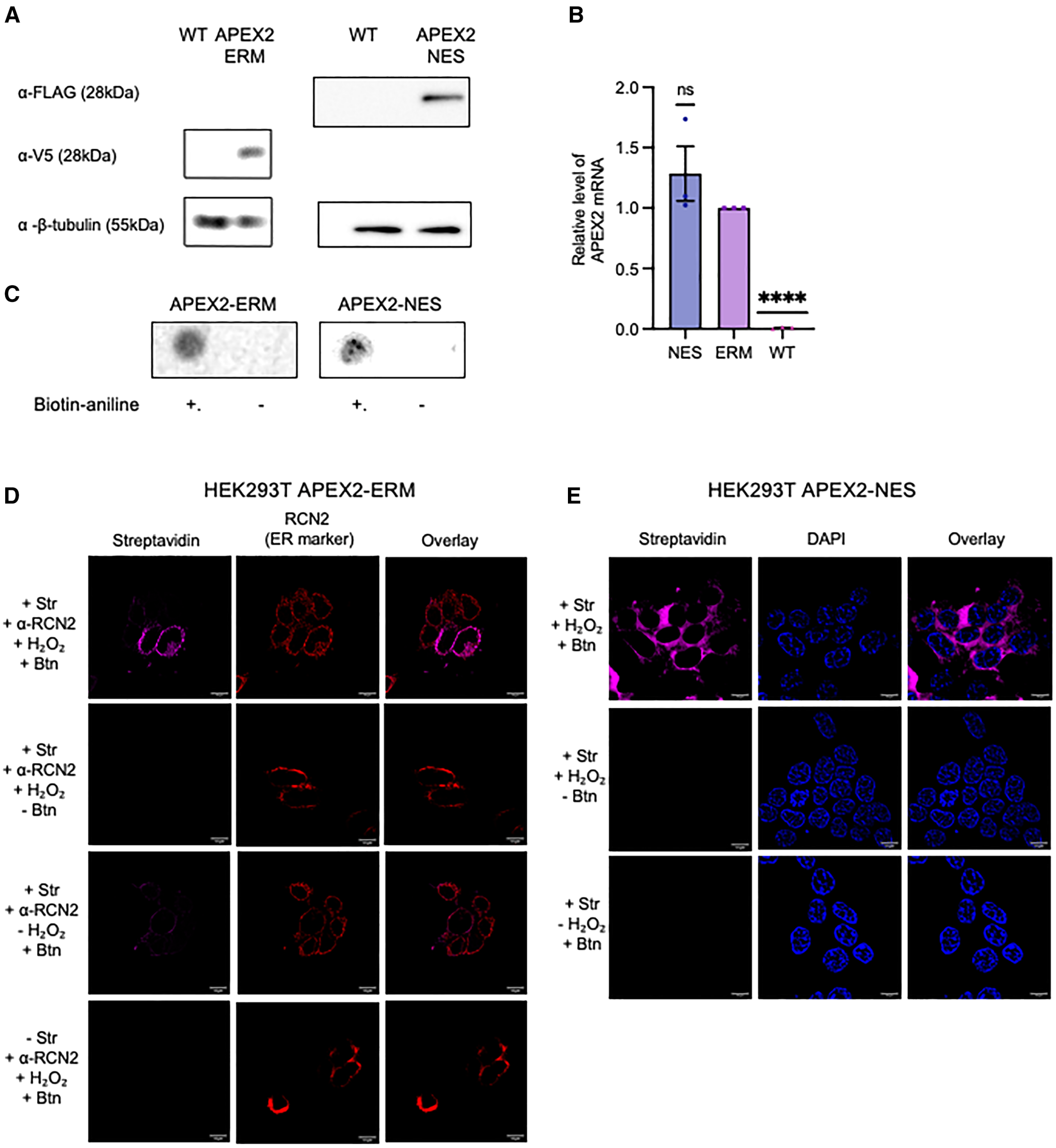

Figure 1. Generation of HEK293T cells with APEX2 localized to ER or cytoplasm

(A) Western blot showing expression of APEX2-ERM (detected by antibody to V5 epitope) or APEX2-NES (detected by antibody to FLAG epitope) in respective HEK293T cell lines generated by lentiviral transduction. Membranes were stripped and re-probed for beta-tubulin as a loading control. Images are representative of three independent experiments. (B) RT-qPCR showing *APEX2* mRNA is expressed at a similar level in both cell lines and is background in WT HEK293T cells. *APEX2* mRNA levels normalized to 18S rRNA are shown relative to the APEX2-ERM cell line. Mean of three independent experiments, error bars indicate SEM, ****$p < 0.0001$, ns = not significant (Student's t test relative to APEX2-ERM). (C) RNA dot blot assay showing APEX2-mediated biotinylation of RNA in both cell lines. Biotin in total RNA extracted from HEK293T APEX2-ERM or APEX2-NES cells following an APEX2 biotinylation reaction was detected by streptavidin-HRP and compared to RNA from a control reaction in which biotin aniline was omitted. APEX2-ERM dot blot is representative of three independent experiments, whereas APEX-NES dot blot is a single experiment. (D) SIM of HEK293T APEX2-ERM cells following an APEX2-mediated biotinylation reaction shows strong overlap between biotin and the ER, detected by streptavidin-AlexaFluor and anti-RCN2 respectively. Manders' colocalization coefficients were $M_1 = 0.8467 \pm 0.1846$ (streptavidin overlap with RCN2) and $M_2 = 0.9521 \pm 0.06747$ (RCN2 overlap with streptavidin), calculated from 24 cells. (E) As (D), except HEK293T APEX2-NES cells were used, and DAPI is shown as a nuclear stain. Biotin signal was excluded from the nucleus. Manders' colocalization coefficients were $M_1 = 0.07563 \pm 0.07382$ (streptavidin overlap with DAPI) and $M_2 = 0.2336 \pm 0.2388$ (DAPI overlap with streptavidin), calculated from 57 cells. Scale bars represent 10 μm.

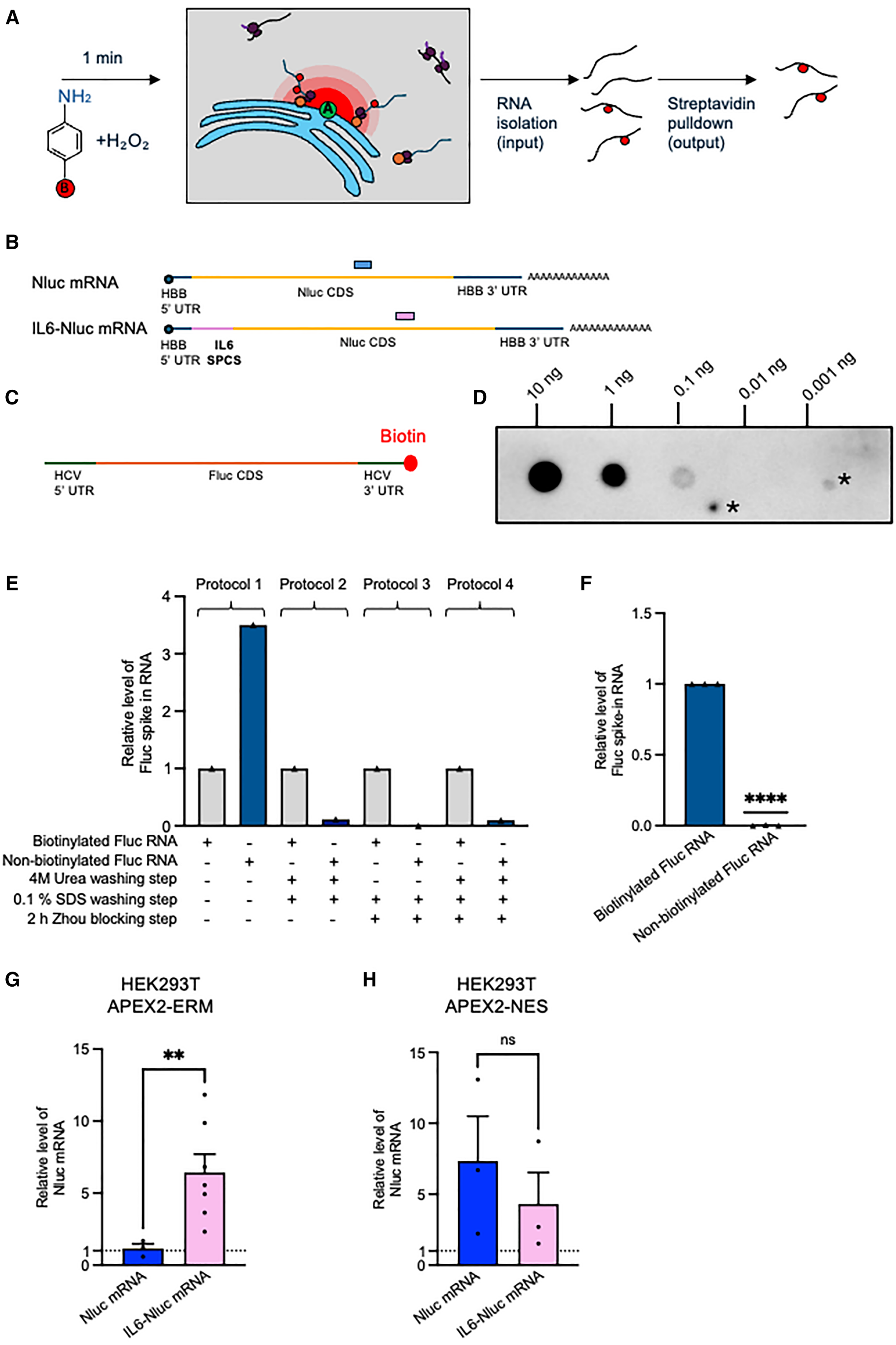

(legend on next page)

mRNA levels were similar in both (Figure 1B). We carried out APEX2 biotinylation by treating cells with biotin aniline, shown to be the most effective substrate for RNA biotinylation,[15] and hydrogen peroxide and confirmed this was effective on total RNA in both cell lines (Figure 1C). Structured illumination microscopy (SIM) showed APEX2 colocalized with the ER in APEX2-ERM cells and was present in the cytoplasm in APEX2-NES cells (Figures S1A and S1B). SIM following an APEX2 biotinylation reaction showed that biotin localization strongly overlapped with the ER in HEK293T APEX2-ERM cells (Figure 1D). In APEX2-NES cells, biotin showed diffuse subcellular distribution outside the nucleus (Figure 1E).

Next, we combined published APEX2 labeling[14] and pull-down[16] methodology with RT-qPCR to investigate delivery of IVT mRNA to the ER or cytoplasm (Figure 2A). IVT Nanoluciferase (*Nluc*) mRNA without a signal peptide coding sequence (SPCS), expected to localize to the cytoplasm (*Nluc* mRNA), was compared with *Nluc* mRNA containing the interleukin 6 (IL6) SPCS (*IL6-Nluc* mRNA) (Figure 2B). The N-terminal IL6 signal peptide is expected to mediate cotranslational trafficking to the ER and synthesis of secreted Nluc protein.[17] Both mRNAs were co-transcriptionally capped and enzymatically polyadenylated and included the hemoglobin subunit beta (*HBB*) 5′ and 3′ untranslated regions (UTRs), which promote efficient translation.[18] Both APEX2 cell lines were transfected with both IVT mRNAs, and biotinylation reactions were carried out after 4 h. However, APEX2-RT-qPCR of *Nluc* mRNA in pull-down relative to input RNA, normalized to a no-biotin control, gave very variable results in both HEK293T APEX2-ERM (Figure S2A) and HEK293T APEX2-NES cells (Figure S2B), possibly because the methodology was previously developed for use with endogenous mRNA and RNA-seq.[14] To improve APEX2-RT-qPCR as a method, we generated a 3′-biotinylated firefly luciferase (*Fluc)* RNA (Figure 2C) and confirmed its detection at ≥0.1 ng by dot blot (Figure 2D). This biotinylated RNA or a non-biotinylated control were applied to streptavidin-conjugated beads using various blocking and washing conditions, based on three published APEXseq methods,[14,16,19] and optimal conditions for pull-down of biotinylated RNA with low background were identified by *Fluc* RT-qPCR (Figure 2E). Importantly, this optimized protocol (protocol 3 in Figure 2E) remained effective in isolation of biotinylated *Fluc* RNA in the presence of excess total RNA (Figure 2F). Next, we combined APEX2-mediated biotinylation in cells transfected with *Nluc* or *IL6-Nluc* mRNA (Figure 2B), using lipofectamine, with the optimized streptavidin pull-down protocol, including the Fluc-biotin spike-in. In APEX2-ERM cells, *IL6-Nluc* mRNA showed ∼6-fold enrichment in streptavidin pull-down from cells treated with biotin aniline compared to a no-biotin control, whereas there was no enrichment of *Nluc* mRNA (Figure 2G), showing that APEX2-mediated biotinylation selectively detects delivered mRNA localization to the ER. In APEX2-NES cells, *Nluc* mRNA appeared enriched in streptavidin pull-down over background, although this was not statistically significant (Figure 2H). However, *IL6-Nluc* mRNA appeared similarly enriched (Figure 2H), indicating that APEX2-mediated biotinylation in APEX2-NES cells is not selective for cytoplasmic mRNA. Our normalization method, relative to a biotinylated spike-in RNA and a matched no-biotin condition, controls for experimental variations in transfection efficiency, streptavidin pull-down, or RNA extraction. However, we also confirmed that levels of both transfected *Nluc* mRNAs in input RNA were comparable (Figures S2C and S2D).

Having shown that our optimized APEX2-RT-qPCR protocol detects IVT mRNA delivery to the ER, we decided to test whether it can be used to investigate the impact of mRNA features on this delivery. We focused on the SPCS as its role is underexplored compared to that of UTRs and the CDS.[18] *PMEL-Nluc* mRNA was therefore generated by replacing the SPCS in *IL6-Nluc* IVT mRNA with that of *PMEL* mRNA (Figure 3A). PMEL was chosen as it is a transmembrane protein with an N-terminal signal peptide distinct to that of IL-6[20] and is relevant to mRNA therapeutics as a source of tumor antigens.[21] Both mRNAs were introduced into HEK293T APEX2-ERM cells using lipofectamine. APEX2-RT-qPCR showed a small, but not statistically significant, enrichment in *PMEL-Nluc* at the ER compared to *IL6-Nluc* mRNA (Figure 3B). Input mRNA levels were comparable (Figure S3). In contrast, secreted Nluc production measured in parallel tended to be lower from *PMEL-Nluc* mRNA than from *IL6-Nluc* mRNA at 1, 2, and 4 h, although this difference was not statistically significant (Figure 3C). To explore this in a different cell line, APEX2-ERM was introduced into A549 lung adenocarcinoma cells. APEX2 expression and ER localization of

Figure 2. Development of an improved APEX2-RT-qPCR methodology using a biotinylated spike-in RNA

(A) Schematic diagram showing APEX2-mediated biotinylation at the ER in cells treated with biotin aniline and hydrogen peroxide, followed by RNA isolation and streptavidin pull-down. Green circle represents APEX2, orange circles represent SRP, ribosomes are shown in purple, and red circles represent biotin. Zone of biotinylation is shown in red. (B) Diagram showing *Nluc* and *IL6-Nluc* IVT mRNAs, expected to be localized to cytoplasm and ER, respectively. (C) Diagram showing the 3′-biotinylated *Fluc* spike-in RNA. (D) RNA dot blot detection of serially diluted quantities of biotinylated spike-in RNA using streptavidin-HRP. *Non-specific background signal. Image is representative of three independent experiments. (E) Biotinylated spike-in *Fluc* RNA, or non-biotinylated control RNA, was bound to and eluted from streptavidin magnetic beads using various block and wash conditions. Following isolation, *Fluc* RNA levels were determined by RT-qPCR, normalized to *Nluc* RNA added as a spike-in during RNA extraction and are shown relative to the biotinylated RNA for each condition. Data represent a single experiment. (F) As (E), except that the optimized conditions in protocol 3 were used, and 5 μg total HEK293T RNA was included, showing that spike-in RNA can be isolated from an excess of non-biotinylated RNA. Data represent mean of three independent experiments, +SEM. ****$p < 0.0001$ (Student's t test relative to biotinylated *Fluc* mRNA). (G) APEX2-mediated biotinylation and streptavidin pull-down were carried out in HEK293T APEX2-ERM cells at 4 h post-transfection with *Nluc* or *IL6-Nluc* mRNA. Following elution, *Nluc* mRNA levels were determined by RT-qPCR, normalized to *Fluc*-biotin RNA included as a spike-in during initial RNA extraction, and expressed relative to the no biotin control for each RNA. Data represent mean of seven (*IL6-Nluc*) or three (*Nluc*) independent experiments, +SEM. **$p < 0.005$ (Student's t test). (H) As (G), except that HEK293T APEX2-NES cells were transfected. Data represent mean of three independent experiments, +SEM. ns = not significant (Student's t test).

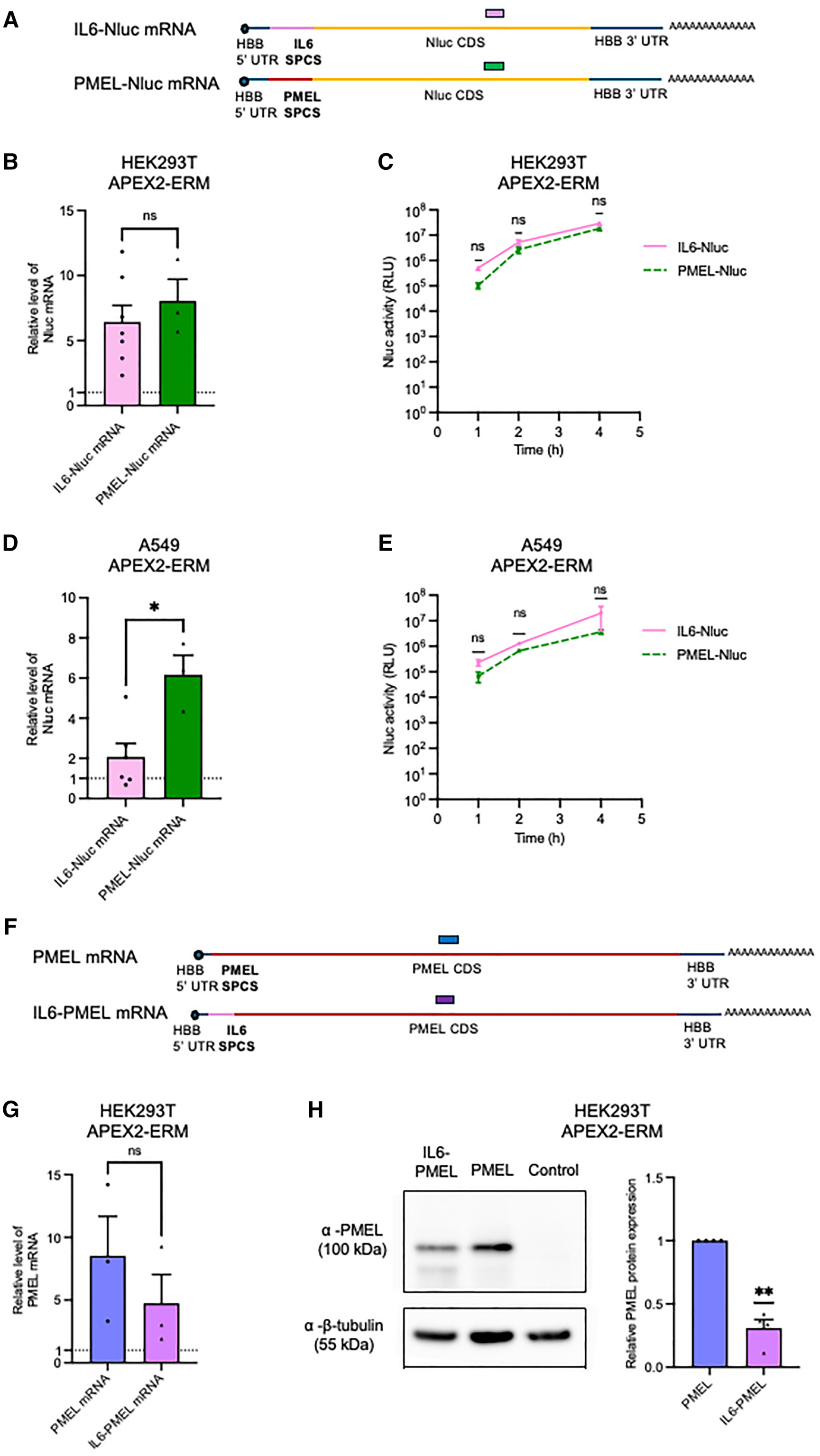

Figure 3. Investigation of determinants of effective ER targeting

(A) Schematic diagram of *IL6-Nluc* and *PMEL-Nluc* mRNAs. (B) APEX2-mediated biotinylation and streptavidin pull-down were carried out in HEK293T APEX2-ERM cells following transfection with *IL6-Nluc* or *PMEL-Nluc* mRNA. Following elution, *Nluc* mRNA levels were determined by RT-qPCR, normalized to *Fluc*-biotin RNA included as a spike-in during initial RNA extraction, and expressed relative to the no biotin control for each RNA. Data represent mean of seven (*IL6-Nluc*) or three (*PMEL-Nluc*) independent experiments, +SEM. ns = not significant (Student's t test). (C) Secreted Nluc assays were carried out on media of the cells transfected in (B) at time points before the biotinylation reaction. Data represent mean of three independent experiments, +SEM. ns = not significant (Student's t test at each time point with Bonferroni correction). (D) As (B), except that A549 APEX2-ERM cells were used. Data represent mean of six (*IL6-Nluc*) or three (*PMEL-Nluc*) independent experiments, +SEM. *$p < 0.05$ (Student's t test). (E) As (C), except that A549 APEX2-ERM cells were used. Data represent mean of three independent experiments, +SEM. ns indicates not significant (Student's t test at each time point with Bonferroni correction). (F) Schematic diagram of *PMEL* and *IL6-PMEL* mRNAs. (G) As (B), except that *PMEL* mRNA and *IL6-PMEL* mRNA were introduced into HEK293T APEX2-ERM cells. Data represent mean of three independent experiments, +SEM. ns = not significant (Student's t test). (H) PMEL protein was detected by western blot following transfection of cells as in (G) or *Nluc* mRNA as a negative control. Beta-tubulin is shown as a loading control. Image is representative of four independent experiments. Graph shows densitometry of PMEL relative to beta-tubulin, +SEM. **$p < 0.005$ (Student's t test relative to PMEL).

biotinylation were confirmed by western blotting (Figure S4A) and SIM (Figure S4B). APEX2-RT-qPCR showed stronger enrichment of PMEL-Nluc mRNA than IL6-Nluc mRNA at the ER in A549 cells (Figure 3D), while input mRNA levels were comparable (Figure S5A). Secreted Nluc production was slightly, but not statistically significantly, higher from *IL6-Nluc* mRNA (Figure 3E), similar to HEK293T cells. Finally, we tested the effect of SPCS substitution in the context of the *PMEL* CDS, using mRNAs including the native *PMEL* SPCS (*PMEL*) or the *IL6* SPCS (*IL6-PMEL*) (Figure 3F). Following transfection of HEK293T APEX2-ERM cells and APEX2-RT-qPCR, *PMEL* mRNA was slightly, but not statistically significantly, more enriched at the ER than *IL6-PMEL* mRNA (Figure 3G). Input mRNA levels were similar (Figure S5B). Western blotting showed that PMEL protein production was significantly lower from *IL6-PMEL* mRNA than *PMEL* mRNA (Figure 3H). Overall, our results suggest that the *PMEL* SPCS directs slightly stronger ER localization at 4 h post-transfection than that of *IL6*, particularly in A549 cells, and that protein production shows the same trend for the *PMEL* but not the *Nluc* CDS.

DISCUSSION

Here, we adapted APEX2-RT-qPCR methodology,[14–16] by inclusion of biotinylated spike-in RNA that allowed both optimization of streptavidin binding conditions and subsequent data normalization (Figures 2E–2H), to provide a novel analytical approach to investigate intracellular delivery of mRNA. We found that our optimized protocol was selective in detecting mRNA localization to the ER (Figure 2G), but less so for mRNA targeted to the cytoplasm (Figure 2H). We used this approach to investigate determinants of successful mRNA delivery to the ER in two cell lines, comparing two SPCSs and two CDSs (Figure 3). The *PMEL* SPCS led to a trend toward increased ER localization compared to the *IL6* SPCS in the context of both *Nluc* and *PMEL* CDSs. PMEL protein production was more efficient from *PMEL* than *IL6-PMEL* mRNA (Figure 3H), in line with mRNA localization at the ER (Figure 3G), which could be due to the increased efficiency of translation at the ER that has previously been observed.[22] In contrast, the *PMEL* SPCS did not increase secreted Nluc protein production (Figures 3C and 3E). This could be due to several factors, such as differences in the time course of mRNA targeting to the ER and completion of protein synthesis, dynamics of both ribosome attachment and detachment from the ER, or differences in the rate of cleavage of different signal peptides that affect the rate of Nluc secretion.[8] Our method provides a basis for further exploration of the interplay between ER association and translation for a broader range of signal peptides in the context of therapeutic mRNAs. It would be particularly interesting to investigate ER delivery of mRNAs containing the therapeutically relevant pseudouridine or N^1-methyl-pseudouridine modifications, as ER-derived membranes were shown to increase *in vitro* translation of full-length protein from mRNAs containing these modifications by reducing ribosome collision.[23]

Although normalization to the biotinylated spike-in and method optimization improved reproducibility, we still observed some variation in data, and different numbers of independent repeats in some experiments influenced statistical comparisons. We found that using fresh reagents, particularly hydrogen peroxide, was important to improve consistency of data. Data variability may have been influenced by the use of pooled cell populations following lentiviral transduction, meaning that APEX2 expression may vary between individual cells. Clonal selection of cell lines might give more consistent results but also risks selection of clones that have other genetic changes that may affect function. We did not confirm the expected cytoplasmic and ER localization of *Nluc* mRNAs by microscopy, although luciferase assays showing that *IL6-Nluc* and *PMEL-Nluc* mRNAs produced secreted protein, and *Nluc* mRNA produced cytoplasmic protein, support their correct localization.

There are several advantages of the APEX-RT-qPCR method for investigating intracellular mRNA delivery. First, it directly detects the mRNA rather than indirectly detecting its products. Second, it does not rely on mRNA labeling, which may interfere with translation, allowing us to measure protein production in parallel to mRNA localization. Third, it can be adapted to investigate mRNA delivery to different subcellular locations.[14] Future adaptations to the methodology could include combination with other proximity labeling methods, as used in the TransitID method to investigate protein transport.[24] Approaches based on HaloTag click chemistry have also shown promise for investigation of intracellular delivery of peptides or antisense oligonucleotides,[25–27] but in contrast to the APEX2 method to detect unmodified mRNA, these methods require chloroalkane conjugation of the delivered material.

In summary, the APEX2-RT-qPCR approach presented here shows potential for future investigation of determinants of successful mRNA delivery to locations within the cell, including testing the efficacy of different vehicles in delivering mRNA to subcellular compartments. As a proof of concept, we focused on using the method to investigate the role of different signal peptides in ER localization of reporter mRNAs and observed differences in efficiency of targeting that will be an important topic of future investigation.

MATERIALS AND METHODS

Cell culture and lentiviral transduction

HEK293T and A549 cells were maintained in high-glucose DMEM (Sigma) supplemented with 10% fetal bovine serum (FBS; Gibco). Lentiviral stocks were generated by transfection of HEK293T cells with the packaging vectors pMD2.9 (Addgene 12259) and psPAX2 (Addgene 12260), with either ERM-APEX2 (Addgene 79055) or APEX2-NES (Addgene 92158) included to target APEX2 to the endoplasmic reticulum or cytoplasm, respectively, using Fugene HD (Promega). Lentiviral stocks were prepared by filtration of the media 48 h after transfection and stored at −80°C. Lentiviral transduction of HEK293T and A549 cells was carried out by addition of lentiviral stock with 8 μg/mL Polybrene (Millipore). Blasticidin (Sigma) was added to cells at 24 h post-transduction at 6 μg/mL, and cells were selected for 1.5 weeks and maintained as a mixed cell population. Blasticidin was routinely added to the resulting APEX2-ERM and

APEX2-NES cells in culture every 1 to 2 weeks to ensure maintenance of the transgene.

Plasmid construction and *in vitro* transcription

The plasmid encoding firefly luciferase (Fluc) flanked by hepatitis C virus (HCV) 5′ and 3′ UTRs and under control of a T7 promoter (p5′LUC3′) has been described previously[28] and was used as a template for synthesis of Fluc spike-in RNA. To generate a plasmid encoding secreted nanoluciferase (Nluc) named pIL6-Nluc-hbb (Addgene 239843), a gBlock (IDT) encoding the IL6-Nluc CDS from pNL2.3 (Promega), flanked by hemoglobin subunit beta (*HBB*) 5′ and 3′UTRs and including an upstream T7 promoter, was designed and the full region amplified by PCR and inserted into the p5′LUC3′ backbone[28] (derived from Promega pGL3Control) in place of the HCV UTR-Fluc cassette by restriction digest. An equivalent plasmid encoding cytoplasmic Nluc, named pNluc-hbb (Addgene 239753), was generated by the same approach using a gBlock with the *IL6* SPCS removed. To generate the PMEL plasmid, named pPMEL-hbb (Addgene 239863), three gBlocks encompassing the full *PMEL* CDS (NCBI NM_006928.5), including SPCS, with a KVP to KGP mutation at the N-terminal previously shown to enhance immunogenicity,[21] were purchased (IDT). The *PMEL* CDS was amplified by PCR from the gBlocks and inserted in place of the *IL6-Nluc* CDS by Gibson assembly (NEBuilder HiFi). Finally, SPCS substitution was carried out by Gibson assembly to generate the plasmids pIL6-PMEL-hbb (Addgene 239868) and pPMEL-Nluc-hbb (Addgene 239862). Templates for *in vitro* transcription (IVT) were generated by either *EcoRI*-mediated linearization or PCR amplification of the plasmid. IVT was then carried out using the T7 Megascript kit (Thermo Fisher) to generate 5′LUC3′ spike-in RNA, which was not capped or adenylated. Capped, polyadenylated RNA was generated from all other templates by IVT using the T7 mMessage mMachine kit (Thermo Fisher), followed by DNase treatment. Polyadenylation was then carried out using the Poly(A) tailing kit (Thermo Fisher). All RNAs were purified using the RNA Clean & Concentrator-25 kit (Zymo), according to the manufacturer's instructions, and stored in aliquots at −80°C. Primer sequences are available in Table S1 and mRNA sequences in Table S2.

pCp biotinylation of spike-in RNA

To generate a biotinylated spike-in RNA, 1 μg 5′LUC3′ IVT RNA was incubated with 0.1 mM pCp-biotin (Jena), 1 mM ATP, 15% PEG8000, 10% DMSO, 1 X T4 RNA ligase buffer, and 10 units T4 RNA ligase 1 (NEB) in a final volume of 30 μL. The reaction was incubated overnight at 16°C, and RNA was purified using the RNA Clean & Concentrator-25 kit (Zymo) to remove unincorporated biotin.

RNA transfection and luciferase assays

IVT RNA was introduced into APEX2-ERM or APEX2-NES cells in 6-well plates using Lipofectamine 2000 (Thermo Fisher), according to the manufacturer's instructions. Following transfection with the secreted nanoluciferase-expressing mRNAs *IL6-Nluc* or *PMEL-Nluc*, 30 μL of culture medium was harvested at 1, 2, and 4 h and assayed using the Nano-Glo Luciferase Assay System (Promega) in a GloMax Navigator luminometer (Promega). APEX2-mediated biotinylation was then carried out on the transfected cells at 4 h post-transfection. For *PMEL* or *IL6-PMEL* IVT mRNA transfection, one well of a 6-well plate was used for APEX2-mediated biotinylation, and a second well transfected in parallel was harvested by lysis in RIPA at 4 h post-transfection for analysis by western blotting.

Western blotting

Protein lysates were harvested from 6-well plates in RIPA buffer and equal masses of total protein separated by SDS-PAGE, before transfer to PVDF membrane (Thermo Fisher) using a TurboBlot (BioRad). Primary antibodies to V5 (Invitrogen 46–0705, 1:50 000), FLAG-M2 (Sigma F1804, 1:5000), and GP100 (Abcam ab137078, 1:5000) were used to detect APEX2-NES, APEX2-ERM, and PMEL, respectively. Membranes were stripped using Re-Blot Plus Strong Solution (Millipore) and re-probed with an antibody to beta-tubulin (Abcam ab6046, 1:5000) as a loading control. HRP-conjugated secondary antibodies to mouse or rabbit IgG were purchased from Sigma and Dako, respectively, and used at 1:1,000. Signal was detected with Pierce ECL substrate (Thermo Fisher) and visualized on an LAS-3000 Imager (FujiFilm). Densitometry was carried out using ImageJ.

APEX2 labeling

APEX2-mediated biotinylation of RNA was carried out as described in Fazal et al.,[14] except that cells were treated with 500 μM biotin aniline (Iris Biotech), as used in,[15] instead of biotin phenol. For streptavidin dot blot or RT-qPCR, total RNA was isolated using TRI reagent (Sigma) immediately following wash steps. Control experiments were carried out in parallel by omission of biotin aniline.

RNA isolation

Total RNA was isolated from cells by direct application of TRI reagent (Sigma) to the plate. Following streptavidin pull-down and elution, RNA was isolated by addition of TRIzol LS (Thermo Fisher) at a 3:1 ratio relative to the RNA-containing solution. For optimization of pull-down conditions, 0.1 ng *Nluc* mRNA was added with TRI reagent or TRIzol LS as a control for efficient RNA isolation. Subsequent isolation was carried out according to the manufacturers' instructions, with addition of 15 μg GlycoBlue (Thermo Fisher) as co-precipitant in TRIzol LS extraction where quantities of RNA were low. RNA was resuspended in 15–40 μL of nuclease-free water.

Optimization of streptavidin pull-down

Streptavidin pull-down was optimized by adaptation of the protocols of[14,16,19] using 0.5 ng 3′-biotinylated 5′LUC3'. Different methods of washing and blocking beads before incubation with RNA, incubation with RNA, washing beads following incubation with RNA, and elution from beads were tested. The final protocol based on these optimization experiments used 10 μL Pierce Streptavidin magnetic beads per pull-down (ThermoFisher), which were washed three times with bead washing buffer (5 mM Tris-HCl pH = 7.5, 0.5 mM EDTA, 1 M

NaCl, 0.1% TWEEN 20), then twice in 0.1M NaOH/0.05 M NaCl, then once in 0.1M NaCl. The beads were then incubated in blocking buffer (2 mg/mL yeast tRNA, 50 μg/mL glycogen), with rotation, for 2 h at room temperature (RT). The beads were then washed three times with 4 M NaCl washing buffer (0.1 M Tris-HCl pH = 7.5, 4 M NaCl, 10 mM EDTA, 0.2% Tween 20), then three times with 1 X binding buffer (0.1 M Tris-HCl pH = 7.5, 1 M NaCl, 10 mM EDTA, 0.2% Tween 20), before resuspension in 100 μL 2 X binding buffer per pull-down. Five hundred nanograms of total RNA from APEX2 biotinylation and 0.1 ng of 3′-biotinylated 5′LUC3′ RNA were then added to the beads, diluted to a total volume of 200 μL in water such that the binding buffer concentration was 1 X, and incubated for 2 h at 4°C. The beads were then washed once with bead washing buffer, twice with 0.1% SDS in PBS, and twice more with bead washing buffer. To elute the RNA, beads were resuspended in 54 μL nuclease-free water. Thirty-three microliters of 3 X proteinase digestion buffer (3 X PBS, 6% N-laurylsarcosine sodium solution, 0.03 mM EDTA, 0.015 M DTT), 10 μL of Proteinase K (20 mg/mL, Thermo Fisher) and 3 μL of RNasin ribonuclease inhibitor (Promega) were added to give a final volume of 100 μL. The beads were incubated at 42°C for 1 h and then 55°C for 1 h with shaking at 400 RPM in a ThermoMixer (Eppendorf). The beads were placed on a magnet, the supernatant was removed, and 25 μL water was added before addition of 375 μL TRIzol LS (Thermo Fisher) to extract eluted RNA.

RT-qPCR

Reverse transcription was carried out using GoScript Reverse Transcription System (Promega) with random primers, according to the manufacturer's instructions. Either 100 ng total RNA or 8 μL of 16 μL RNA eluted from streptavidin beads was used in the reaction. qPCR was performed in technical triplicate using GoTaq (Promega) in a Rotor-Gene Q (Qiagen). Primer sequences are available in Table S1.

Streptavidin RNA dot blot assay

To detect biotinylation of either spike-in RNA or APEX2-biotinylated RNA, a dot blot assay was used. Equal masses of total RNA following APEX labeling, or serial dilutions of pCp-biotin-labeled 5′LUC3′ spike-in RNA, were made up to 2 μL and spotted onto Amersham Hybond N+ membrane (GE Healthcare), allowed to air dry, and UV-crosslinked twice using the standard 1,200 J setting on a Stratalinker (Stratagene). The membrane was then probed following the protocol in,[29] except that 1:2,500 Streptavidin-HRP (Abcam) was used. Signal was detected with Pierce ECL substrate (Thermo Fisher) and visualized on an LAS-3000 Imager (FujiFilm).

Structured illumination microscopy

Cells were seeded in 24 well plates on 1.5 H precision coverslips (Marienfeld), pre-coated in Poly-D-lysine (Gibco). Cells were fixed in 4% paraformaldehyde in PBS for 10 min at RT and permeabilized in 0.5% saponin from Quillaja Bark (Sigma) for 10 min at RT, washed three times with ice-cold PBS, and blocked in 0.1% saponin/3% BSA in PBS for 1 h at RT. Primary antibodies were diluted in blocking solution and incubated with coverslips at 4°C overnight. APEX2-NES or APEX2-ERM were detected using primary antibodies to V5 and FLAG-M2 as described for western blotting, used at 1:400 and 1:200, respectively. Anti-RCN2 (Proteintech 10193-2-AP, 1:200) was used to detect the endoplasmic reticulum. Secondary antibodies were anti-mouse Alexa Fluor 488 (Invitrogen A28175) and anti-rabbit Alexa Fluor 546 (Invitrogen A11071) at 1:500. Where relevant, cytoplasm was stained using Alexa Fluor 546 Phalloidin (Invitrogen A22283, 1:500) at the same time as secondary antibody. Nuclei were stained using DAPI (Invitrogen) at 0.1 μg/mL for 15 min at RT in the dark after secondary antibody. Following APEX2-mediated labeling, biotin was detected using Streptavidin Alexa Fluor 488 (Invitrogen S11223) or Streptavidin Alexa Fluor 647 (Invitrogen S21374) at 1:500. Coverslips were washed three times in ice-cold PBS, dipped in water, and mounted in 8 μL Fluoromount G containing 1:100 0.1 μm TetraSpeck beads (Invitrogen). Coverslips were allowed to harden overnight at RT and stored in the dark prior to imaging using the Elyra PS1 Super Resolution system. A 63× objective lens was used with 30°C oil to match the refractive index.

Data analysis

RT-qPCR data were analyzed using the $2^{-\Delta\Delta Ct}$ method relative to 18S rRNA for *APEX2* mRNA. For optimization of pull-down conditions using *Fluc* biotinylated spike-in RNA, normalization was relative to *Nluc* mRNA added as a spike-in during RNA isolation. For biotinylation experiments, normalization was relative to the *Fluc* biotinylated spike-in RNA. Statistical analysis was carried out using Student's t test with Welch's correction, or one sample Student's t test when data were shown relative to a standardized control, using GraphPad Prism software. For luciferase data, Bonferroni correction for multiple comparisons was included to account for the multiple timepoints measured.

SIM images were analyzed using Manders' Colocalization Coefficients using ZEN Black software (Zeiss).

DATA AVAILABILITY

All data are presented within the manuscript and supplemental information. Plasmids generated as part of this study have been submitted to Addgene.

ACKNOWLEDGMENTS

We are grateful to Robert Markus and Seema Bagia in the School of Life Sciences Imaging Facility (SLIM), University of Nottingham, for helpful advice and assistance with microscopy. We would like to thank Alexander Kondrashov (School of Medicine, University of Nottingham) for advice on generation of biotinylated spike-in RNA. We thank Federico Dajas-Bailador and Alex Rathbone (School of Life Sciences, University of Nottingham) for provision of cell culture facilities during a programmed lab closure. This work was funded by the United Kingdom Research and Innovation (UKRI) Engineering and Physical Sciences Research Council (EPSRC) grant EP/S023054/1 (PhD studentships to A.D.S. and M.E.H.) and UKRI Biotechnology and Biological Science Research Council (BBSRC) grant BB/W01713X/1 to C.L.J.

AUTHOR CONTRIBUTIONS

C.L.J. designed and supervised the project and wrote the manuscript. A.D.S. carried out experimental work and contributed to project design and manuscript preparation. M.E.H. carried out experimental work. A.D.R.V. contributed to project supervision and

experimental work. N.H. and S.S. contributed to project supervision and design and manuscript preparation.

DECLARATION OF INTERESTS

The authors do not have any conflicts of interest.

SUPPLEMENTAL INFORMATION

Supplemental information can be found online at https://doi.org/10.1016/j.omtn.2025.102602.

REFERENCES

1. Rohner, E., Yang, R., Foo, K.S., Goedel, A., and Chien, K.R. (2022). Unlocking the promise of mRNA therapeutics. Nat. Biotechnol. *40*, 1586–1600.
2. von der Haar, T., Mulroney, T.E., Hedayioglu, F., Kurusamy, S., Rust, M., Lilley, K.S., Thaventhiran, J.E., Willis, A.E., and Smales, C.M. (2023). Translation of in vitro-transcribed RNA therapeutics. Front. Mol. Biosci. *10*, 1128067.
3. Deprey, K., Batistatou, N., and Kritzer, J.A. (2020). A critical analysis of methods used to investigate the cellular uptake and subcellular localization of RNA therapeutics. Nucleic Acids Res. *48*, 7623–7639.
4. Liang, F., Lindgren, G., Lin, A., Thompson, E.A., Ols, S., Röhss, J., John, S., Hassett, K., Yuzhakov, O., Bahl, K., et al. (2017). Efficient Targeting and Activation of Antigen-Presenting Cells In Vivo after Modified mRNA Vaccine Administration in Rhesus Macaques. Mol. Ther. *25*, 2635–2647.
5. Das, S., Vera, M., Gandin, V., Singer, R.H., and Tutucci, E. (2021). Intracellular mRNA transport and localized translation. Nat. Rev. Mol. Cell Biol. *22*, 483–504.
6. Zhang, Y., Zhai, S., Huang, H., Qin, S., Sun, M., Chen, Y., Lan, X., Li, G., Huang, Z., Wang, D., et al. (2024). Efficient signal sequence of mRNA vaccines enhances the antigen expression to expand the immune protection against viral infection. J. Nanobiotechnology *22*, 295.
7. Höpfler, M., and Hegde, R.S. (2023). Control of mRNA fate by its encoded nascent polypeptide. Mol. Cell *83*, 2840–2855.
8. Liaci, A.M., and Förster, F. (2021). Take me home, protein roads: Structural insights into signal peptide interactions during ER translocation. Int. J. Mol. Sci. *22*, 11871.
9. Qin, W., Cho, K.F., Cavanagh, P.E., and Ting, A.Y. (2021). Deciphering molecular interactions by proximity labeling. Nat. Methods *18*, 133–143.
10. Choi, C.-R., and Rhee, H.-W. (2022). Proximity labeling: an enzymatic tool for spatial biology. Trends Biotechnol. *40*, 145–148.
11. Roux, K.J., Kim, D.I., Raida, M., and Burke, B. (2012). A promiscuous biotin ligase fusion protein identifies proximal and interacting proteins in mammalian cells. J. Cell Biol. *196*, 801–810.
12. Branon, T.C., Bosch, J.A., Sanchez, A.D., Udeshi, N.D., Svinkina, T., Carr, S.A., Feldman, J.L., Perrimon, N., and Ting, A.Y. (2018). Efficient proximity labeling in living cells and organisms with TurboID. Nat. Biotechnol. *36*, 880–887.
13. Lam, S.S., Martell, J.D., Kamer, K.J., Deerinck, T.J., Ellisman, M.H., Mootha, V.K., and Ting, A.Y. (2015). Directed evolution of APEX2 for electron microscopy and proximity labeling. Nat. Methods *12*, 51–54.
14. Fazal, F.M., Han, S., Parker, K.R., Kaewsapsak, P., Xu, J., Boettiger, A.N., Chang, H. Y., and Ting, A.Y. (2019). Atlas of Subcellular RNA Localization Revealed by APEX-Seq. Cell *178*, 473–490.e26.
15. Zhou, Y., Wang, G., Wang, P., Li, Z., Yue, T., Wang, J., and Zou, P. (2019). Expanding APEX2 Substrates for Proximity-Dependent Labeling of Nucleic Acids and Proteins in Living Cells. Angew. Chem. Int. Ed. Engl. *58*, 11763–11767.
16. Padrón, A., Iwasaki, S., and Ingolia, N.T. (2019). Proximity RNA Labeling by APEX-Seq Reveals the Organization of Translation Initiation Complexes and Repressive RNA Granules. Mol. Cell *75*, 875–887.e5.
17. Hall, M.P., Unch, J., Binkowski, B.F., Valley, M.P., Butler, B.L., Wood, M.G., Otto, P., Zimmerman, K., Vidugiris, G., Machleidt, T., et al. (2012). Engineered Luciferase Reporter from a Deep Sea Shrimp Utilizing a Novel Imidazopyrazinone Substrate. ACS Chem. Biol. *7*, 1848–1857.
18. Leppek, K., Byeon, G.W., Kladwang, W., Wayment-Steele, H.K., Kerr, C.H., Xu, A. F., Kim, D.S., Topkar, V.V., Choe, C., Rothschild, D., et al. (2022). Combinatorial optimization of mRNA structure, stability, and translation for RNA-based therapeutics. Nat. Commun. *13*, 1536.
19. Li, R., Zou, Z., Wang, W., and Zou, P. (2022). Metabolic incorporation of electron-rich ribonucleosides enhances APEX-seq for profiling spatially restricted nascent transcriptome. Cell Chem. Biol. *29*, 1218–1231.e8.
20. Mitchell, S.M., Graham, M., Liu, X., and Leonhardt, R.M. (2021). Identification of critical amino acid residues in the regulatory N-terminal domain of PMEL. Sci. Rep. *11*, 7730.
21. van Stipdonk, M.J.B., Badia-Martinez, D., Sluijter, M., Offringa, R., van Hall, T., and Achour, A. (2009). Design of Agonistic Altered Peptides for the Robust Induction of CTL Directed towards H-2Db in Complex with the Melanoma-Associated Epitope gp100. Cancer Res. *69*, 7784–7792.
22. Voigt, F., Zhang, H., Cui, X.A., Triebold, D., Liu, A.X., Eglinger, J., Lee, E.S., Chao, J. A., and Palazzo, A.F. (2017). Single-Molecule Quantification of Translation-Dependent Association of mRNAs with the Endoplasmic Reticulum. Cell Rep. *21*, 3740–3753.
23. Svitkin, Y.V., Gingras, A.-C., and Sonenberg, N. (2022). Membrane-dependent relief of translation elongation arrest on pseudouridine- and N 1-methyl-pseudouridine-modified mRNAs. Nucleic Acids Res. *50*, 7202–7215.
24. Qin, W., Cheah, J.S., Xu, C., Messing, J., Freibaum, B.D., Boeynaems, S., Taylor, J.P., Udeshi, N.D., Carr, S.A., and Ting, A.Y. (2023). Dynamic mapping of proteome trafficking within and between living cells by TransitID. Cell *186*, 3307–3324.e30.
25. Deprey, K., Batistatou, N., Debets, M.F., Godfrey, J., VanderWall, K.B., Miles, R.R., Shehaj, L., Guo, J., Andreucci, A., Kandasamy, P., et al. (2022). Quantitative Measurement of Cytosolic and Nuclear Penetration of Oligonucleotide Therapeutics. ACS Chem. Biol. *17*, 348–360.
26. Peraro, L., Deprey, K.L., Moser, M.K., Zou, Z., Ball, H.L., Levine, B., and Kritzer, J.A. (2018). Cell Penetration Profiling Using the Chloroalkane Penetration Assay. J. Am. Chem. Soc. *140*, 11360–11369.
27. Peier, A., Ge, L., Boyer, N., Frost, J., Duggal, R., Biswas, K., Edmondson, S., Hermes, J.D., Yan, L., Zimprich, C., et al. (2021). NanoClick: A High Throughput, Target-Agnostic Peptide Cell Permeability Assay. ACS Chem. Biol. *16*, 293–309.
28. Roberts, A.P.E., Lewis, A.P., and Jopling, C.L. (2011). miR-122 activates hepatitis C virus translation by a specialized mechanism requiring particular RNA components. Nucleic Acids Res. *39*, 7716–7729.
29. Makino, S., Kawamata, T., Iwasaki, S., and Ohsumi, Y. (2021). Selectivity of mRNA degradation by autophagy in yeast. Nat. Commun. *12*, 2316.

OMTN, Volume *36*

Supplemental information

A proximity-labeling-based approach to directly detect mRNA delivery to specific subcellular locations

Alfredo D. Smart, Merryn E. Hughes, Angela Downie Ruiz Velasco, Naoto Hori, Snow Stolnik, and Catherine L. Jopling

Supplemental Methods

APEX2-RT-qPCR without spike-in normalization

HEK293T APEX2-ERM or HEK293T APEX2-NES cells were transfected with Nluc or IL6-Nluc mRNA and APEX2 labelling carried out as described in the main methods. In early experiments before optimization of streptavidin pulldown or introduction of a biotinylated spike-in RNA, streptavidin pulldown was carried out as described in.[16] Nluc mRNA levels in pulldown RNA were measured by RT-qPCR relative to 2% input RNA, and are shown relative to a no-biotin control. As the Shapiro-Wilk test showed that data were not normally distributed, statistical comparison was carried out by Mann-Whitney test.

Table S1: Primer sequences. Primers used in PCR amplification for restriction cloning or Gibson assembly to generate plasmids, PCR amplification to generate *in vitro* transcription templates, and for RT-qPCR.

Primer name	Primer sequence 5'-3'	Purpose
HBB_Nluc_F	GATCGTCAAAGCTTACTTGGTC	Restriction cloning of Nluc and IL6-Nluc
HBB_Nluc_R	GATCTGACGAATTCTTGCAATG	
Nluc_alt_frag_F	CAGACACCATGGATCTGGTGCT	Gibson assembly of PMEL-Nluc
Nluc_alt_frag_R	TGTGAAGACTGTAGCCCCCACAGCCAG	
Amp_F	GCCATTGCTACAGGCATCGTGGTGTCACGC	
Nluc_alt_Vec_R	CAGATCCATGGTGTCTGTTTGAGGTTG	
Nluc_alt_Vec_F	GGGCTACAGTCTTCACACTCGAAGATTTCGT	
Amp_R	CTGTAGCAATGGCAACAACGTTGCGC	
PMEL_5'end_F	ACAGACACCATGGATCTGGTGCTAAAAAGATGCC	Gibson assembly of PMEL
PMEL_5'end_R	CACATAGCTCCGGGATCCCCGGC	
PMEL_mid_F	ATCCCGGAGCTATGTGCCTCTTGCTCATTCC	
PMEL_mid_R	CATACCTGTAGCCTCTGGAGTTGACATCTCTG	
PMEL_3'end_F	CCAGAGGCTACAGGTATGACACCTGCAGAGG	
PMEL_3'end_R	AAGCGAGCTCAGACCTGCTGCCCACTGA	
Amp_F	GCCATTGCTACAGGCATCGTGGTGTCACGC	
PMEL_Vec_R	CAGATCCATGGTGTCTGTTTGAGGT	
PMEL_Vec_F	GGTCTGAGCTCGCTTTCTTGC	
Amp_R	CTGTAGCAATGGCAACAACGTTGCGC	
OPMEL_IL6_Frag_F	CAGACACCATGGACTCCTTCTCC	Gibson assembly of IL6-PMEL
OPMEL_IL6_Frag_R	GGGCCTTTTGGGGCAGGGAAGGCA	
Amp_F	GCCATTGCTACAGGCATCGTGGTGTCACGC	
OPMEL_IL6_Vec_R	GGAGTCCATGGTGTCTGTTTGAGGTTGC	
OPMEL_IL6_Vec_F	TGCCCCAAAAGGCCCCAGAAACCAGG	
Amp_R	CTGTAGCAATGGCAACAACGTTGCGC	

HBB_IVT_F	GCCGCTAATACGACTCACTAT	IVT template
HBB_IVT_R	TTGCAATGAAAATAAATGTTTTTTATTAGGCAGAATCCAGA	
APEX_F	TTCGGAACCATCAAGCACCC	RT-qPCR of APEX2 mRNA
APEX_R	CCAGGGTGGAATGGAACCTTAG	
18S_F	CAGCCACCCGAGATTGAGCA	RT-qPCR of 18S rRNA
18S_R	TAGTAGCGACGGGCGGTGTG	
Nluc_F	CCGTATGAAGGTCTGAGCGG	RT-qPCR of Nluc mRNA
Nluc_R	TCTTTTTGCCGTCGAACACG	
Fluc_F	CTAAGGAAGTCGGGGAAGCG	RT-qPCR of Fluc mRNA
Fluc_R	ATCCCCCTCGGGTGTAATCA	
PMEL_F	TTAAGGCTGGTGAAGAGACAAG	RT-qPCR of PMEL mRNA
PMEL_R	AGGATCTCGGCACTTTCAATAC	

Table S2: mRNA sequences. Sequences of Nluc and PMEL mRNAs are shown in 5'-3' direction, with HBB 5' and 3'UTRs in lower case, coding sequence in upper case, and signal peptide coding sequence in bold. All mRNAs also included a 5' cap and a 3' enzymatically added pA tail.

**Nluc mRNA**
gggacatttgcttctgacacaactgtgttcactagcaacctcaaacagacaccATGGTCTTCACACTCG<br>AAGATTTCGTTGGGGACTGGCGACAGACAGCCGGCTACAACCTGGACCAAGTCCTTGAACAGGGAGGTG<br>TGTCCAGTTTGTTTCAGAATCTCGGGGTGTCCGTAACTCCGATCCAAAGGATTGTCCTGAGCGGTGAAA<br>ATGGGCTGAAGATCGACATCCATGTCATCATCCCGTATGAAGGTCTGAGCGGCGACCAAATGGGCCAGA<br>TCGAAAAAATTTTTAAGGTGGTGTACCCTGTGGATGATCATCACTTTAAGGTGATCCTGCACTATGGCA<br>CACTGGTAATCGACGGGGTTACGCCGAACATGATCGACTATTTCGGACGGCCGTATGAAGGCATCGCCG<br>TGTTCGACGGCAAAAAGATCACTGTAACAGGGACCCTGTGGAACGGCAACAAAATTATCGACGAGCGCC<br>TGATCAACCCCGACGGCTCCCTGCTGTTCCGAGTAACCATCAACGGAGTGACCGGCTGGCGGCTGTGCG<br>AACGCATTCTGGCGTAAgctcgctttcttgctgtccaatttctattaaaggttcctttgttccctaagt<br>ccaactactaaactgggggatattatgaagggccttgagcatctggattctgcctaataaaaaacattt<br>attttcattgcaa
**IL6-Nluc mRNA**
gggacatttgcttctgacacaactgtgttcactagcaacctcaaacagacacc**ATGGACTCCTTCTCCA**<br>**CAAGCGCCTTCGGTCCAGTTGCCTTCTCCCTGGGCCTGCTCCTGGTGTTGCCTGCTGCCTTCCCTGCCC**<br>**CA**GTCTTCACACTCGAAGATTTCGTTGGGGACTGGCGACAGACAGCCGGCTACAACCTGGACCAAGTCC<br>TTGAACAGGGAGGTGTGTCCAGTTTGTTTCAGAATCTCGGGGTGTCCGTAACTCCGATCCAAAGGATTG<br>TCCTGAGCGGTGAAAATGGGCTGAAGATCGACATCCATGTCATCATCCCGTATGAAGGTCTGAGCGGCG<br>ACCAAATGGGCCAGATCGAAAAAATTTTTAAGGTGGTGTACCCTGTGGATGATCATCACTTTAAGGTGA<br>TCCTGCACTATGGCACACTGGTAATCGACGGGGTTACGCCGAACATGATCGACTATTTCGGACGGCCGT<br>ATGAAGGCATCGCCGTGTTCGACGGCAAAAAGATCACTGTAACAGGGACCCTGTGGAACGGCAACAAAA<br>TTATCGACGAGCGCCTGATCAACCCCGACGGCTCCCTGCTGTTCCGAGTAACCATCAACGGAGTGACCG<br>GCTGGCGGCTGTGCGAACGCATTCTGGCGTAAgctcgctttcttgctgtccaatttctattaaaggttc

ctttgttccctaagtccaactactaaactgggggatattatgaagggccttgagcatctggattctgcc
taataaaaaacatttattttcattgcaa

PMEL-Nluc mRNA

gggacatttgcttctgacacaactgtgttcactagcaacctcaaacagacacc**ATGGATCTGGTGCTAA**
AAAGATGCCTTCTTCATTTGGCTGTGATAGGTGCTTTGCTGGCTGTGGGGGCTACAGTCTTCACACTCG
AAGATTTCGTTGGGGACTGGCGACAGACAGCCGGCTACAACCTGGACCAAGTCCTTGAACAGGGAGGTG
TGTCCAGTTTGTTTCAGAATCTCGGGGTGTCCGTAACTCCGATCCAAAGGATTGTCCTGAGCGGTGAAA
ATGGGCTGAAGATCGACATCCATGTCATCATCCCGTATGAAGGTCTGAGCGGCGACCAAATGGGCCAGA
TCGAAAAAATTTTTAAGGTGGTGTACCCTGTGGATGATCATCACTTTAAGGTGATCCTGCACTATGGCA
CACTGGTAATCGACGGGGTTACGCCGAACATGATCGACTATTTCGGACGGCCGTATGAAGGCATCGCCG
TGTTCGACGGCAAAAAGATCACTGTAACAGGGACCCTGTGGAACGGCAACAAAATTATCGACGAGCGCC
TGATCAACCCCGACGGCTCCCTGCTGTTCCGAGTAACCATCAACGGAGTGACCGGCTGGCGGCTGTGCG
AACGCATTCTGGCGTAAgctcgctttcttgctgtccaatttctattaaaggttcctttgttccctaagt
ccaactactaaactgggggatattatgaagggccttgagcatctggattctgcctaataaaaaacattt
attttcattgcaa

PMEL mRNA

gggacatttgcttctgacacaactgtgttcactagcaacctcaaacagacacc**ATGGATCTGGTGCTAA**
AAAGATGCCTTCTTCATTTGGCTGTGATAGGTGCTTTGCTGGCTGTGGGGGCTACAAAAGGCCCCAGAA
ACCAGGACTGGCTTGGTGTCTCAAGGCAACTCAGAACCAAAGCCTGGAACAGGCAGCTGTATCCAGAGT
GGACAGAAGCCCAGAGACTTGACTGCTGGAGAGGTGGTCAAGTGTCCCTCAAGGTCAGTAATGATGGGC
CTACACTGATTGGTGCAAATGCCTCCTTCTCTATTGCCTTGAACTTCCCTGGAAGCCAAAAGGTATTGC
CAGATGGGCAGGTTATCTGGGTCAACAATACCATCATCAATGGGAGCCAGGTGTGGGGAGGACAGCCAG
TGTATCCCCAGGAAACTGACGATGCCTGCATCTTCCCTGATGGTGGACCTTGCCCATCTGGCTCTTGGT
CTCAGAAGAGAAGCTTTGTTTATGTCTGGAAGACCTGGGGCCAATACTGGCAAGTTCTAGGGGGCCCAG
TGTCTGGGCTGAGCATTGGGACAGGCAGGGCAATGCTGGGCACACACACCATGGAAGTGACTGTCTACC
ATCGCCGGGGATCCCGGAGCTATGTGCCTCTTGCTCATTCCAGCTCAGCCTTCACCATTACTGACCAGG
TGCCTTTCTCCGTGAGCGTGTCCCAGTTGCGGGCCTTGGATGGAGGGAACAAGCACTTCCTGAGAAATC
AGCCTCTGACCTTTGCCCTCCAGCTCCATGACCCCAGTGGCTATCTGGCTGAAGCTGACCTCTCCTACA
CCTGGGACTTTGGAGACAGTAGTGGAACCCTGATCTCTCGGGCACTTGTGGTCACTCATACTTACCTGG
AGCCTGGCCCAGTCACTGCCCAGGTGGTCCTGCAGGCTGCCATTCCTCTCACCTCCTGTGGCTCCTCCC
CAGTTCCAGGCACCACAGATGGGCACAGGCCAACTGCAGAGGCCCCTAACACCACAGCTGGCCAAGTGC
CTACTACAGAAGTTGTGGGTACTACACCTGGTCAGGCGCCAACTGCAGAGCCCTCTGGAACCACATCTG
TGCAGGTGCCAACCACTGAAGTCATAAGCACTGCACCTGTGCAGATGCCAACTGCAGAGAGCACAGGTA
TGACACCTGAGAAGGTGCCAGTTTCAGAGGTCATGGGTACCACACTGGCAGAGATGTCAACTCCAGAGG
CTACAGGTATGACACCTGCAGAGGTATCAATTGTGGTGCTTTCTGGAACCACAGCTGCACAGGTAACAA
CTACAGAGTGGGTGGAGACCACAGCTAGAGAGCTACCTATCCCTGAGCCTGAAGGTCCAGATGCCAGCT
CAATCATGTCTACGGAAAGTATTACAGGTTCCCTGGGCCCCCTGCTGGATGGTACAGCCACCTTAAGGC
TGGTGAAGAGACAAGTCCCCCTGGATTGTGTTCTGTATCGATATGGTTCCTTTTCCGTCACCCTGGACA
TTGTCCAGGGTATTGAAAGTGCCGAGATCCTGCAGGCTGTGCCGTCCGGTGAGGGGGATGCATTTGAGC
TGACTGTGTCCTGCCAAGGCGGGCTGCCCAAGGAAGCCTGCATGGAGATCTCATCGCCAGGGTGCCAGC
CCCCTGCCCAGCGGCTGTGCCAGCCTGTGCTACCCAGCCCAGCCTGCCAGCTGGTTCTGCACCAGATAC
TGAAGGGTGGCTCGGGGACATACTGCCTCAATGTGTCTCTGGCTGATACCAACAGCCTGGCAGTGGTCA
GCACCCAGCTTATCATGCCTGGTCAAGAAGCAGGCCTTGGGCAGGTTCCGCTGATCGTGGGCATCTTGC
TGGTGTTGATGGCTGTGGTCCTTGCATCTCTGATATATAGGCGCAGACTTATGAAGCAAGACTTCTCCG
TACCCCAGTTGCCACATAGCAGCAGTCACTGGCTGCGTCTACCCCGCATCTTCTGCTCTTGTCCCATTG
GTGAGAACAGCCCCCTCCTCAGTGGGCAGCAGGTCTGAgctcgctttcttgctgtccaatttctattaa

aggttcctttgttccctaagtccaactactaaactgggggatattatgaagggccttgagcatctggat
tctgcctaataaaaaacatttattttcattgcaa

IL6-PMEL mRNA

gggacatttgcttctgacacaactgtgttcactagcaacctcaaacagacacc**ATGGACTCCTTCTCCA**
CAAGCGCCTTCGGTCCAGTTGCCTTCTCCCTGGGCCTGCTCCTGGTGTTGCCTGCTGCCTTCCCTGCCC
CAAAAGGCCCCAGAAACCAGGACTGGCTTGGTGTCTCAAGGCAACTCAGAACCAAAGCCTGGAACAGGC
AGCTGTATCCAGAGTGGACAGAAGCCCAGAGACTTGACTGCTGGAGAGGTGGTCAAGTGTCCCTCAAGG
TCAGTAATGATGGGCCTACACTGATTGGTGCAAATGCCTCCTTCTCTATTGCCTTGAACTTCCCTGGAA
GCCAAAAGGTATTGCCAGATGGGCAGGTTATCTGGGTCAACAATACCATCATCAATGGGAGCCAGGTGT
GGGGAGGACAGCCAGTGTATCCCCAGGAAACTGACGATGCCTGCATCTTCCCTGATGGTGGACCTTGCC
CATCTGGCTCTTGGTCTCAGAAGAGAAGCTTTGTTTATGTCTGGAAGACCTGGGGCCAATACTGGCAAG
TTCTAGGGGGCCCAGTGTCTGGGCTGAGCATTGGGACAGGCAGGGCAATGCTGGGCACACACACCATGG
AAGTGACTGTCTACCATCGCCGGGGATCCCGGAGCTATGTGCCTCTTGCTCATTCCAGCTCAGCCTTCA
CCATTACTGACCAGGTGCCTTTCTCCGTGAGCGTGTCCCAGTTGCGGGCCTTGGATGGAGGGAACAAGC
ACTTCCTGAGAAATCAGCCTCTGACCTTTGCCCTCCAGCTCCATGACCCCAGTGGCTATCTGGCTGAAG
CTGACCTCTCCTACACCTGGGACTTTGGAGACAGTAGTGGAACCCTGATCTCTCGGGCACTTGTGGTCA
CTCATACTTACCTGGAGCCTGGCCCAGTCACTGCCCAGGTGGTCCTGCAGGCTGCCATTCCTCTCACCT
CCTGTGGCTCCTCCCCAGTTCCAGGCACCACAGATGGGCACAGGCCAACTGCAGAGGCCCCTAACACCA
CAGCTGGCCAAGTGCCTACTACAGAAGTTGTGGGTACTACACCTGGTCAGGCGCCAACTGCAGAGCCCT
CTGGAACCACATCTGTGCAGGTGCCAACCACTGAAGTCATAAGCACTGCACCTGTGCAGATGCCAACTG
CAGAGAGCACAGGTATGACACCTGAGAAGGTGCCAGTTTCAGAGGTCATGGGTACCACACTGGCAGAGA
TGTCAACTCCAGAGGCTACAGGTATGACACCTGCAGAGGTATCAATTGTGGTGCTTTCTGGAACCACAG
CTGCACAGGTAACAACTACAGAGTGGGTGGAGACCACAGCTAGAGAGCTACCTATCCCTGAGCCTGAAG
GTCCAGATGCCAGCTCAATCATGTCTACGGAAAGTATTACAGGTTCCCTGGGCCCCCTGCTGGATGGTA
CAGCCACCTTAAGGCTGGTGAAGAGACAAGTCCCCCTGGATTGTGTTCTGTATCGATATGGTTCCTTTT
CCGTCACCCTGGACATTGTCCAGGGTATTGAAAGTGCCGAGATCCTGCAGGCTGTGCCGTCCGGTGAGG
GGGATGCATTTGAGCTGACTGTGTCCTGCCAAGGCGGGCTGCCCAAGGAAGCCTGCATGGAGATCTCAT
CGCCAGGGTGCCAGCCCCCTGCCCAGCGGCTGTGCCAGCCTGTGCTACCCAGCCCAGCCTGCCAGCTGG
TTCTGCACCAGATACTGAAGGGTGGCTCGGGGACATACTGCCTCAATGTGTCTCTGGCTGATACCAACA
GCCTGGCAGTGGTCAGCACCCAGCTTATCATGCCTGGTCAAGAAGCAGGCCTTGGGCAGGTTCCGCTGA
TCGTGGGCATCTTGCTGGTGTTGATGGCTGTGGTCCTTGCATCTCTGATATATAGGCGCAGACTTATGA
AGCAAGACTTCTCCGTACCCCAGTTGCCACATAGCAGCAGTCACTGGCTGCGTCTACCCCGCATCTTCT
GCTCTTGTCCCATTGGTGAGAACAGCCCCCTCCTCAGTGGGCAGCAGGTCTGAgctcgctttcttgctg
tccaatttctattaaaggttcctttgttccctaagtccaactactaaactgggggatattatgaagggc
cttgagcatctggattctgcctaataaaaaacatttattttcattgcaa

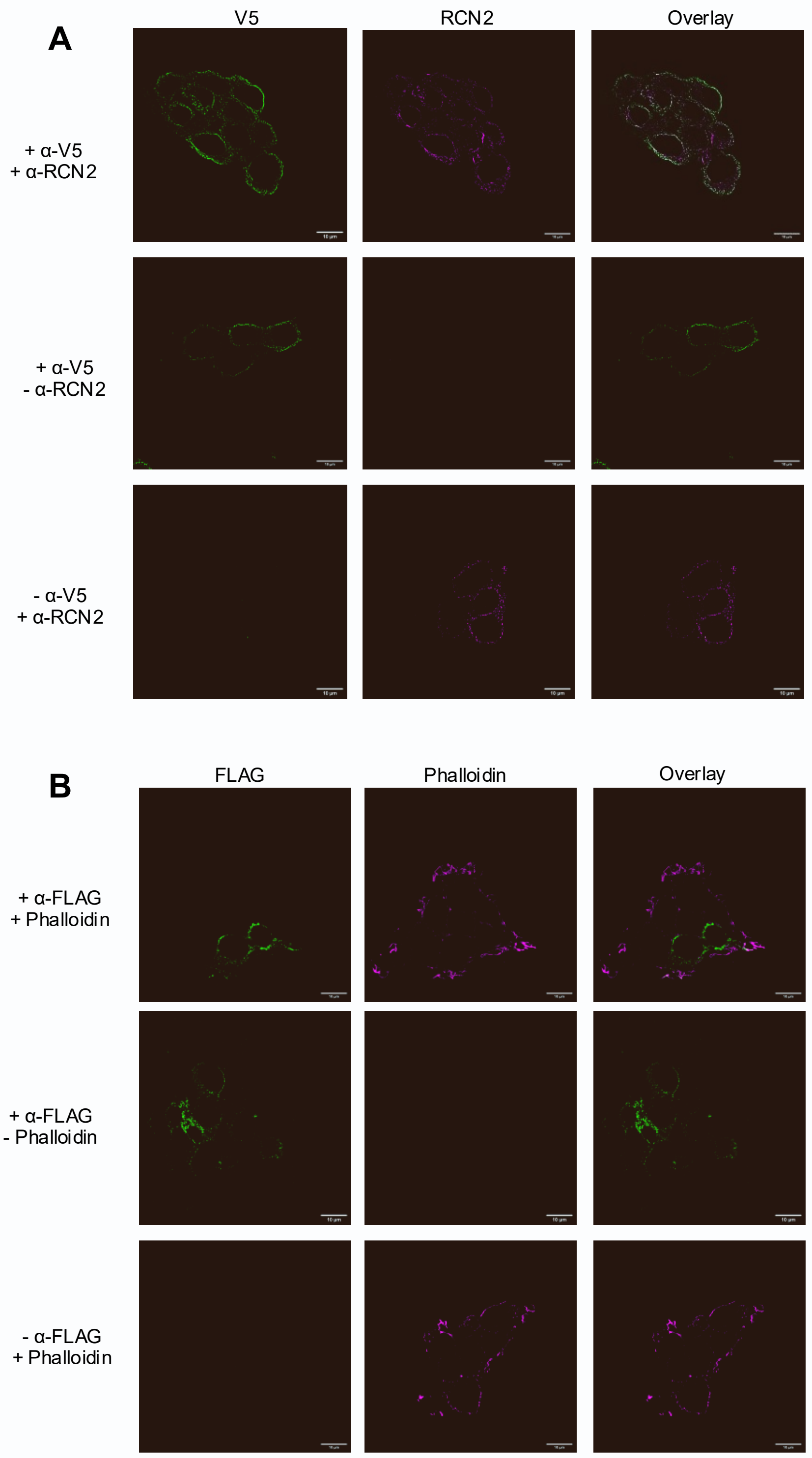

Figure S1. APEX2 localization in HEK293T APEX2-ERM and APEX2-NES cells. (A) SIM of HEK293T APEX2-ERM cells shows overlap between APEX2 and the ER, detected with antibodies to the V5 epitope and RCN2 respectively. Manders' Colocalization Coefficients were $M_1 = 0.6233 \pm 0.04986$ (V5 overlap with RCN2), $M_2 = 0.6701 \pm 0.08678$ (RCN2 overlap with V5), calculated from 17 cells. (B) SIM of HEK293T APEX2-NES cells shows some overlap between APEX2 and filamentous actin as a cytoplasmic marker, detected by an antibody to the FLAG epitope and phalloidin, respectively. Manders' Colocalization Coefficients were $M_1 = 0.6989 \pm 0.1855$ (FLAG overlap with phalloidin), $M_2 = 0.1120 \pm 0.1141$ (phalloidin overlap with FLAG), calculated from 15 cells. Scale bars represent 10 μm.

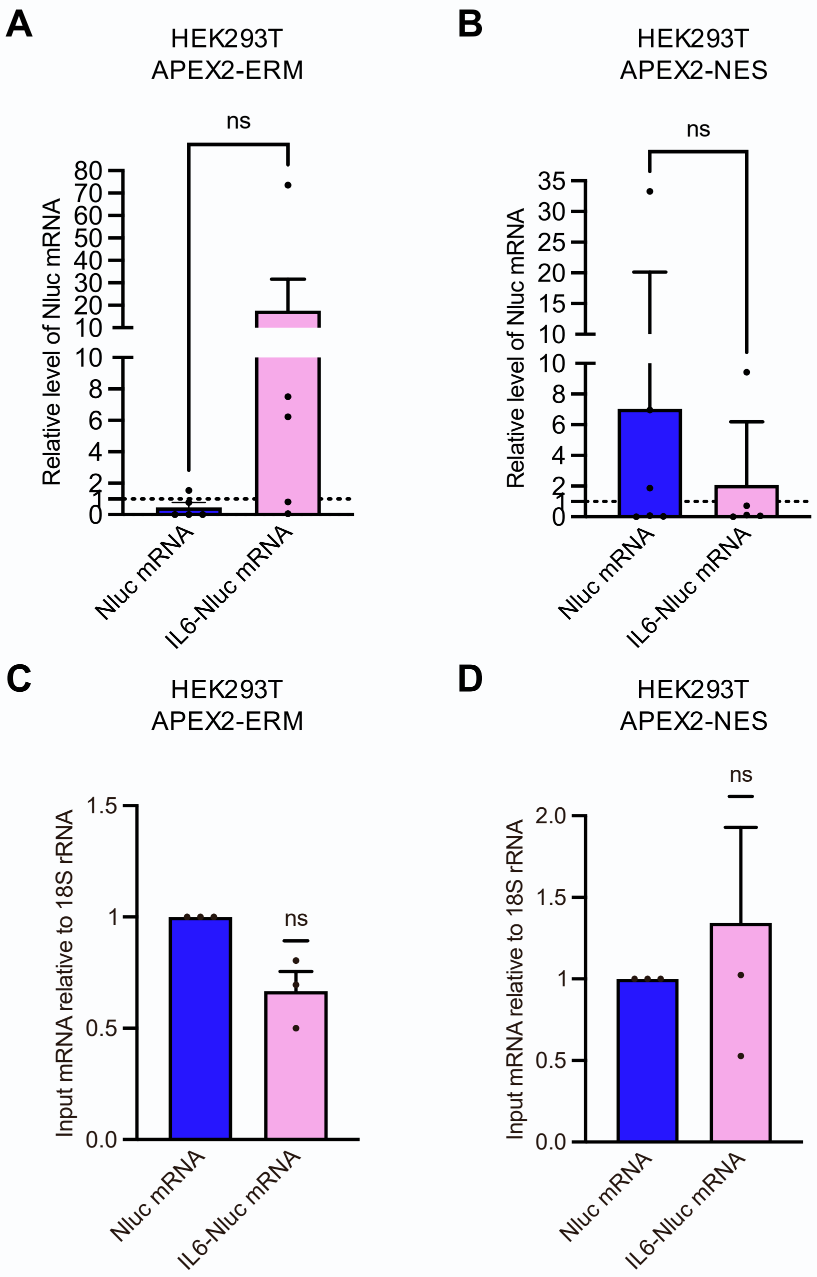

Figure S2. APEX2-RT-qPCR comparison of Nluc mRNA delivery to ER and cytoplasm. (A) HEK293T APEX2-ERM cells were transfected with Nluc or IL6-Nluc mRNA and APEX2 biotinylation and streptavidin pulldown carried out without inclusion of a spike-in RNA. Nluc mRNA in the pulldown measured by RT-qPCR was normalized to 2% input RNA and shown relative to a no-biotin control. Data represent mean of 5 independent experiments, +SEM. ns=not significant (Mann-Whitney test comparison of Nluc and IL6-Nluc). (B) As (A), except that HEK293T APEX2-NES cells were used. Data represent mean of 6 (Nluc) and 5 (IL6-Nluc) independent experiments, +SEM. ns=not significant (Mann-Whitney test comparison of Nluc and IL6-Nluc). (C) HEK293T APEX2-ERM cells were transfected with Nluc or IL6-Nluc mRNA for experiments shown in Figure 2G. Nluc mRNA was measured by RT-qPCR in input mRNA before streptavidin pulldown and show relative to 18S rRNA. Data represent mean of 3 independent experiments, +SEM. ns=not significant (Student's t test comparison of Nluc and IL6-Nluc). (D) As (C), except that HEK293T APEX2-NES cells were transfected for experiment shown in Figure 2H. Data represent mean of 3 independent experiments, +SEM. ns=not significant (Student's t test comparison of Nluc and IL6-Nluc).

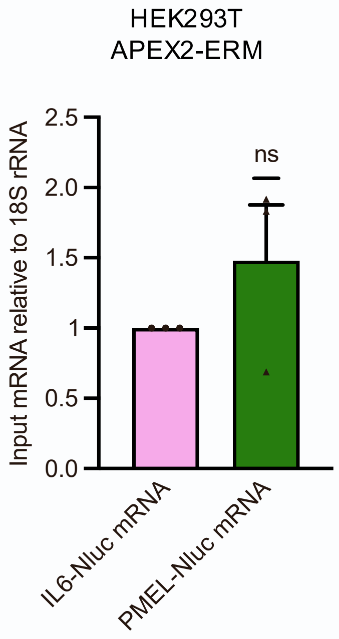

Figure S3. Input mRNA levels for HEK293T APEX2-ERM pulldown experiment. (A) HEK293T APEX2-ERM cells were transfected with IL6-Nluc or PMEL-Nluc mRNA for experiments shown in Figure 3B. Nluc mRNA was measured by RT-qPCR in input mRNA before streptavidin pulldown and show relative to 18S rRNA. Data represent mean of 3 independent experiments, +SEM. ns=not significant (Student's t test comparison of IL6-Nluc and PMEL-Nluc).

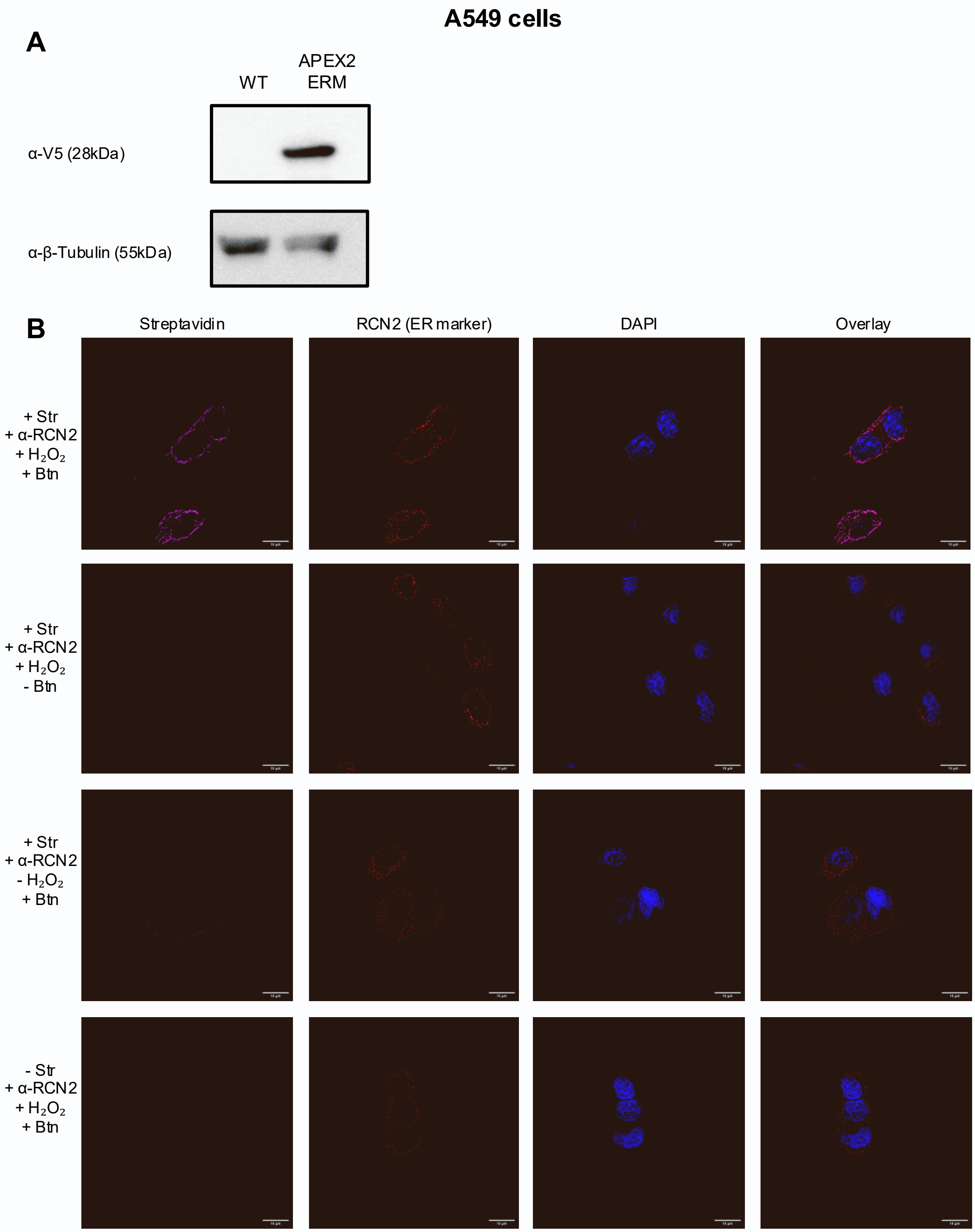

Figure S4. Characterization of A549 APEX2-ERM cells. (A) Western blot showing expression of APEX2-ERM (detected by antibody to V5 epitope) in an A549 cell line generated by lentiviral transduction. The membrane was stripped and re-probed for β-tubulin as a loading control. Image is representative of n=3 independent experiments. (B) SIM of HEK293T APEX2-ERM cells following an APEX2-mediated biotinylation reaction shows overlap between biotin and the ER, detected by streptavidin-AlexaFluor and anti-RCN2 respectively. DAPI is included as a nuclear stain. Manders' Colocalization Coefficients were $M_1 = 0.5584 \pm 0.05264$ (streptavidin overlap with RCN2), $M_2 = 0.8908 \pm 0.08908$ (RCN2 overlap with streptavidin), calculated from 25 cells. Scale bars represent 10 μm.

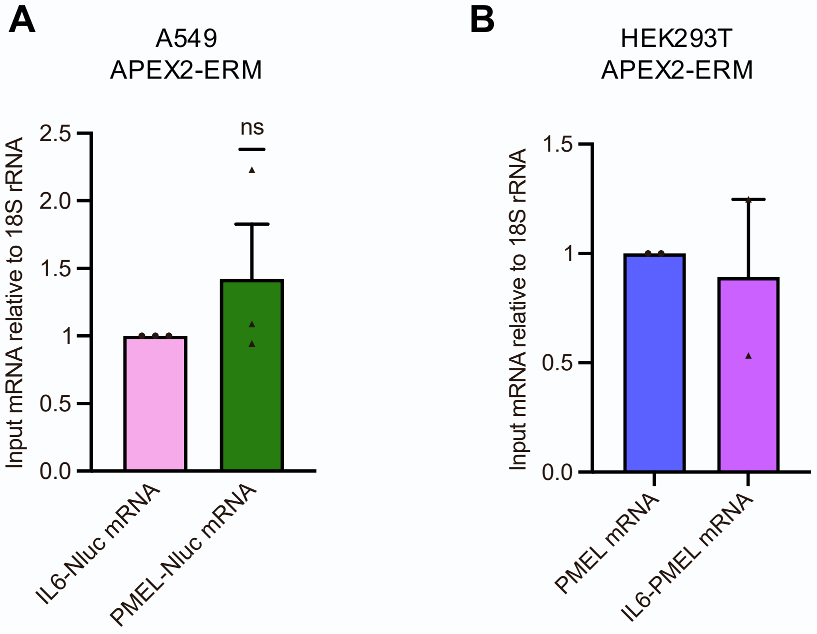

Figure S5. Input mRNA levels for APEX2 pulldown experiments. (A) A549 APEX2-ERM cells were transfected with IL6-Nluc or PMEL-Nluc mRNA for the experiments shown in Figure 3D. Nluc mRNA was measured by RT-qPCR in input mRNA before streptavidin pulldown and show relative to 18S rRNA. Data represent mean of 3 independent experiments, +SEM. ns=not significant (Student's t test comparison of IL6-Nluc and PMEL-Nluc). (B) As (A), except that HEK293T APEX2-ERM cells were transfected with PMEL or IL6-PMEL mRNA for the experiments shown in Figure 3G. Data represent mean of 2 independent experiments +SEM.
